# Estrogen Mediated-Activation of miR-191/425 Cluster Modulates Tumorigenicity of Breast Cancer Cells Depending on Estrogen Receptor Status

**DOI:** 10.1371/journal.pgen.1003311

**Published:** 2013-03-07

**Authors:** Gianpiero Di Leva, Claudia Piovan, Pierluigi Gasparini, Apollinaire Ngankeu, Cristian Taccioli, Daniel Briskin, Douglas G. Cheung, Brad Bolon, Laura Anderlucci, Hansjuerg Alder, Gerard Nuovo, Meng Li, Marilena V. Iorio, Marco Galasso, Santhanam Ramasamy, Guido Marcucci, Danilo Perrotti, Kimerly A. Powell, Anna Bratasz, Michela Garofalo, Kenneth P. Nephew, Carlo M. Croce

**Affiliations:** 1 Department of Molecular Virology, Immunology, and Medical Genetics, School of Medicine, The Ohio State University, Columbus, Ohio, United States of America; 2 Department of Experimental Oncology, Start Up Unit, Istituto Nazionale Tumori, Fondazione IRCCS, Milano, Italy; 3 Department of Cancer Biology, Cancer Institute “Paul O'Gorman,” University College of London, London, United Kingdom; 4 Comparative Pathology and Mouse Phenotyping Shared Resource, College of Veterinary Medicine, The Ohio State University, Columbus, Ohio, United States of America; 5 Dipartimento di Scienze Statistiche, Facoltà di Scienze Statistiche, Università di Bologna, Bologna, Italy; 6 Medical Sciences Program, School of Medicine, Indiana University, Bloomington, Indiana, United States of America; 7 Dipartimento di Morfologia ed Embriologia and LTTA, University of Ferrara, Ferrara, Italy; Fred Hutchinson Cancer Research Center, United States of America

## Abstract

MicroRNAs (miRNAs), single-stranded non-coding RNAs, influence myriad biological processes that can contribute to cancer. Although tumor-suppressive and oncogenic functions have been characterized for some miRNAs, the majority of microRNAs have not been investigated for their ability to promote and modulate tumorigenesis. Here, we established that the miR-191/425 cluster is transcriptionally dependent on the host gene, DALRD3, and that the hormone 17β-estradiol (estrogen or E2) controls expression of both miR-191/425 and DALRD3. MiR-191/425 locus characterization revealed that the recruitment of estrogen receptor α (ERα) to the regulatory region of the miR-191/425-DALRD3 unit resulted in the accumulation of miR-191 and miR-425 and subsequent decrease in DALRD3 expression levels. We demonstrated that miR-191 protects ERα positive breast cancer cells from hormone starvation-induced apoptosis through the suppression of tumor-suppressor EGR1. Furthermore, enforced expression of the miR-191/425 cluster in aggressive breast cancer cells altered global gene expression profiles and enabled us to identify important tumor promoting genes, including SATB1, CCND2, and FSCN1, as targets of miR-191 and miR-425. Finally, *in vitro* and *in vivo* experiments demonstrated that miR-191 and miR-425 reduced proliferation, impaired tumorigenesis and metastasis, and increased expression of epithelial markers in aggressive breast cancer cells. Our data provide compelling evidence for the transcriptional regulation of the miR-191/425 cluster and for its context-specific biological determinants in breast cancers. Importantly, we demonstrated that the miR-191/425 cluster, by reducing the expression of an extensive network of genes, has a fundamental impact on cancer initiation and progression of breast cancer cells.

## Author Summary

MicroRNAs are small noncoding RNAs that act as posttranscriptional repressors of gene expression. A pivotal role for miRNAs in all the molecular processes driving initiation and progression of various malignancies, including breast cancer, has been described. Divergent miRNA expression between normal and neoplastic breast tissues has been demonstrated, as well as differential miRNA expression among the molecular subtypes of breast cancer. Over half of all breast cancers overexpress ERα, and several studies have shown that miRNA expression is controlled by ERα. We assessed the global change in microRNA expression after estrogen starvation and stimulation in breast cancer cells and identified that miR-191/425 and the host gene DALRD3 are positively associated to ERα-positive tumors. We demonstrated that ERα regulates the miR-191/425 cluster and verified the existence of a transcriptional network that allows a dual effect of estrogen on miR-191/425 and their host gene. We show that estrogen induction of miR-191/425 supports *in vitro* and *in vivo* the estrogen-dependent proliferation of ERα positive breast cancer cells. On the contrary, miR-191/425 cluster reprograms gene expression to impair tumorigenicity and metastatic potential of highly aggressive ERα negative breast cancer cells.

## Introduction

MicroRNAs (miRNAs) are a class of evolutionarily conserved regulatory RNAs that pleiotropically suppress gene expression at post-transcriptional level [1]. MiRNAs control the expression of 10–30% of the human transcriptome and are crucial regulators of both physiologic and pathologic processes [2]–[4]. In cancer, the spectrum of miRNAs expressed in neoplastic cells differs dramatically from that found in normal cells and it is now well established that miRNAs play fundamental roles in essentially all aspects of tumor biology [5], [6].

In breast cancer, divergent miRNA expression between normal and neoplastic tissues has been demonstrated, as well as differential miRNA expression among the molecular subtypes of breast cancer, including luminal A, luminal B, Her2+ and basal-like [7], [8]. MiRNAs have been shown to play an important role in breast cancer initiation and progression. For example, overexpression of miR-21 in breast carcinomas has been shown to target important tumor-suppressor genes such as PTEN, PDCD4, and TPM1, and was associated with advanced clinical stage, lymph node metastasis, and poor patient prognosis [9], [10]. MiR-10a was reported to be overexpressed in about 50% of metastatic breast cancer and transcriptionally activated by the pro-metastatic transcription factor TWIST1 [11]. Reduced expression of miR-126 and miR-335 in the majority of primary breast tumors from relapsed patients was reported, and simultaneous loss of miR-126 and miR-335 expression was associated with poor distal metastasis-free survival [12]. Oncogene regulation by miRNAs has also been reported, including tyrosine kinase receptors HER-2 and HER-3 by miR-125b and miR-205, respectively [13], [14], and the miR-200 family, known to reduce cell migration and invasiveness by targeting ZEB transcription factor members, was suppressed in metastatic breast cancer [15], [16].

miRNA regulation by estrogen receptor-alpha (ERα), the most important prognostic and therapeutic indicator in breast cancer, has recently been described by us and others [17]–[20]. Specifically, the majority of miRNAs upregulated by ERα are key components of a negative feedback loop that restrict E2 action and thus play a tumor suppressive role. In this regard, ERα-activation of let-7 family members limits the expression of oncogenes, such as Ras and c-Myc, and promotes differentiation of cancer cells [18]; ERα-mediated activation of the miR-17/92 cluster functions as a tumor suppressing mechanism in breast cancer through the downregulation of cyclin D1 and AIB1 by the miR-17/20/106 family and the direct suppression of ERα mediated by miR-18 and miR-19 [19]. We and others have described a double-negative feedback loop involving E2-suppressed microRNAs that target ERα, specifically miR-206 and miR-221&222, resulting in upregulation of ERα expression and low miRNA level in luminal A-type breast cancers [17], [21].

Recent works from our group have shown that miR-191 is highly induced in several human solid tumors including colon, lung, pancreas, prostate, and stomach cancer [22], as well as acute lymphocytic leukemia (ALL)-associated hematopoietic malignancies [23]. We have also reported a strong positive correlation between miR-191 expression and ERα levels in breast tumors [7], suggesting an oncogenic function for this miR. A role for miR-191 in tumorigenesis is further strengthened by several findings, including that miR-191 is induced by a dioxin family carcinogen, the miR is hypomethylated and overexpressed in liver cancer [24], [25], and miR-191 inhibition decreases cell proliferation and tumor growth of hepatocellular carcinoma cells [24]. Furthermore, miR-191 overexpression promotes cell growth and suppresses apoptosis of gastric cancer cells [26]. However, in ovarian and thyroid follicular cancer, miR-191 represses MDM4 or CDK6 expression, respectively, thereby delaying cancer progression and tumor-related death [27], [28]. These contradictory findings indicate that the precise role for miR-191 in human neoplasia may be tumor-type specific and not well understood.

In this current study, we report a positive association between ERα expression and miR-191 and miR-425, two intronic miRNAs hosted by the putative protein coding gene DALR anticodon binding domain containing 3 (DALRD3), and further show direct control of the miR-191/425/DALRD3 transcriptional unit by the E2/ERα axis. We evaluated that the estrogen dependent activation of miR-191/425 induces proliferation in part by targeting the estrogen modulated tumor-suppressor gene, EGR1. We also demonstrated that, when constitutively expressed in highly aggressive ERα negative breast cancer cells, the miR-191/425 cluster reprograms gene expression to impair tumorigenicity and metastatic potential through the suppression of several different oncogenic proteins.

## Results

### miR191/425 cluster is positively correlated with ERα levels

MiR-191 and miR-425 are highly conserved miRNAs found on human chromosome 3 within the first intron of DALRD3 (Figure S1). Given their genomic organization and proximity, we hypothesized that miR-191 and miR-425 are co-transcribed and transcriptionally dependent on the host gene DALRD3. We examined expression of mature miR-191, miR-425, and DALRD3 mRNA in 20 different normal human tissues using qRT-PCR (Figure S2A). Both miRNAs were detected in all tissues and, their levels of expression were highly correlated, as shown by scatter plot analyses, (R^2^ = 0.7351; p<0.001) (Figure S2B). However, only a partial correlation was observed between the host gene DALRD3 and miR-191 (R^2^ = 0.4058; p<0.001) or miR-425 (R^2^ = 0.2101; p<0.001) (Figure S2B), suggesting the existence of DALRD3-independent mechanism of miR-191/425 expression/accumulation in some tissues.

**Figure 1:**
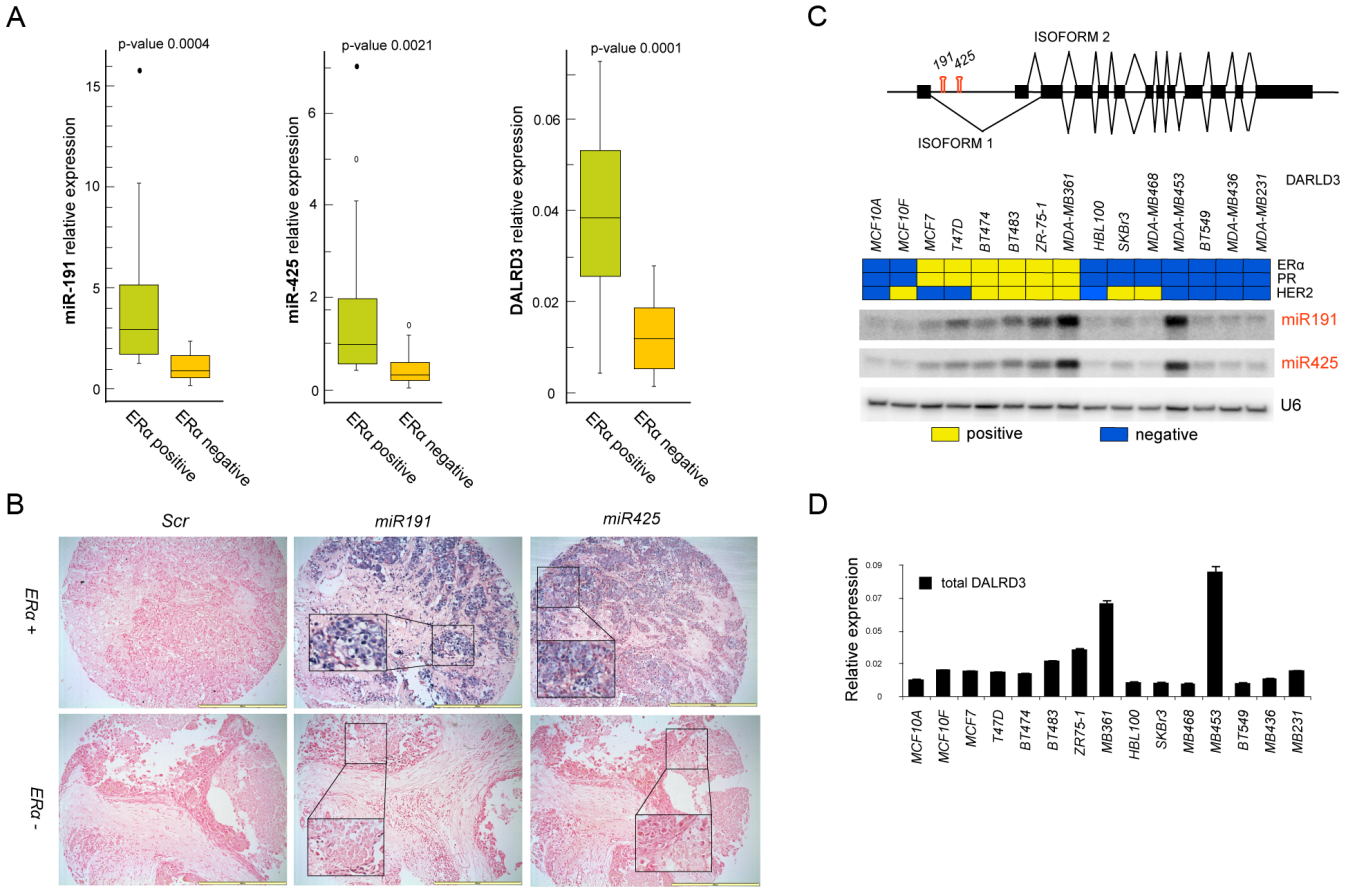
miR-191/425 expression is associated with ERα. (A) Expression of miR-191, miR-425 and DALRD3 mRNA in 44 breast tumor specimens with different status of estrogen receptor α (20 ER− and 24 ER+) (p-value<0.001). (B) In-situ hybridization for miR-191 and miR-425 and scrambled control probe (SCR) on breast cancer tissue array containing 132 cores of breast cancer tissues. Bars are 200 µm and inserts represent higher magnification (20×) of selected areas. (C) Schematic representation of miR-191 and miR-425 location in the first intron of the putative protein coding gene DALRD3 with its two different isoforms. (D) Heat map representing the status of ERα, progesterone receptor (PR) and HER2, confirmed by western blot analyses, in a set of 15 breast cancer cell lines (Yellow: positive; Blue: negative). Northern blot analyses depicting miR-191 and miR-425 expression levels. snRNAU6 levels were used as a loading control. Expression levels of all isoforms of DALRD3 mRNA were determined by Taqman qRT-PCR. All error bars represent mean s.d.

Based on the previous association between miR-191 and ERα and the miR-191 and miR-425 co-expression results (Figure S2A), it was of interest to examine ERα positive breast tumors for the expression of miR-191 and miR-425. qRT-PCR analysis of 44 human breast cancer specimens with different ERα status revealed that miR-191 and miR-425 expression was higher (p-value<0.01) in ERα positive than ERα negative tumors (Figure 1A). DALRD3 mRNA also showed a significant positive correlation with the ERα status (Figure 1A and Figure S3A). Next, to further verify the positive association between ERα levels and miR-191/425 expression, miRNA in-situ hybridization was performed on an independent set of 132 human breast cancer specimens. As anticipated, the majority of ERα positive breast tumors were also miR-191 (80%) and miR-425 (87%) positive, while only 23% and 15% of ERα negative specimens expressed miR-191 and miR-425, respectively (Figure 1B and Figure S4A). Furthermore, co-labeling of miR-191 and miR-425 by miRNA in situ-hybridization on the same ERα positive breast specimens showed co-localization of the two microRNAs in the majority of breast tumor cells (Figure S4B).

Finally, a set of 16 different breast cancer cells, clustered by ERα, progesterone receptor (PR) and HER2 expression was also analyzed for the expression of miR-191, miR-425 and the host gene DALRD3. Expression of both miR-191 and miR-425 was higher in the ERα positive cell lines, with the exception of MDA-MB-453 (a non-aggressive ERα negative/androgen receptor positive breast cancer cell line but with a gene expression profile that overlaps with ERα positive breast cancer cells [29]) (Figure 1C, 1D). DALRD3 expression correlated with the expression levels of the mature miRNAs (R^2^ = 0.725 for miR-191, p<0.01; R^2^ = 0.63 for miR-425, p<0.01) (Figure 1C, 1D and Figure S2C). Moreover, we assessed the expression levels of the two different alternative splicing variants of DALRD3 and confirmed that the two variants are both transcribed and their expression levels are higher in the ERα positive than ERα negative breast cancer cells (Figure S3B). Taken together, these data revealed for the first time that miR-191 and miR-425 are co-transcribed and preferentially expressed in ERα positive breast cancer cells and tumors.

### Estrogen-dependent duality of miR191/425-DALRD3 transcriptional unit

**Figure 2:**
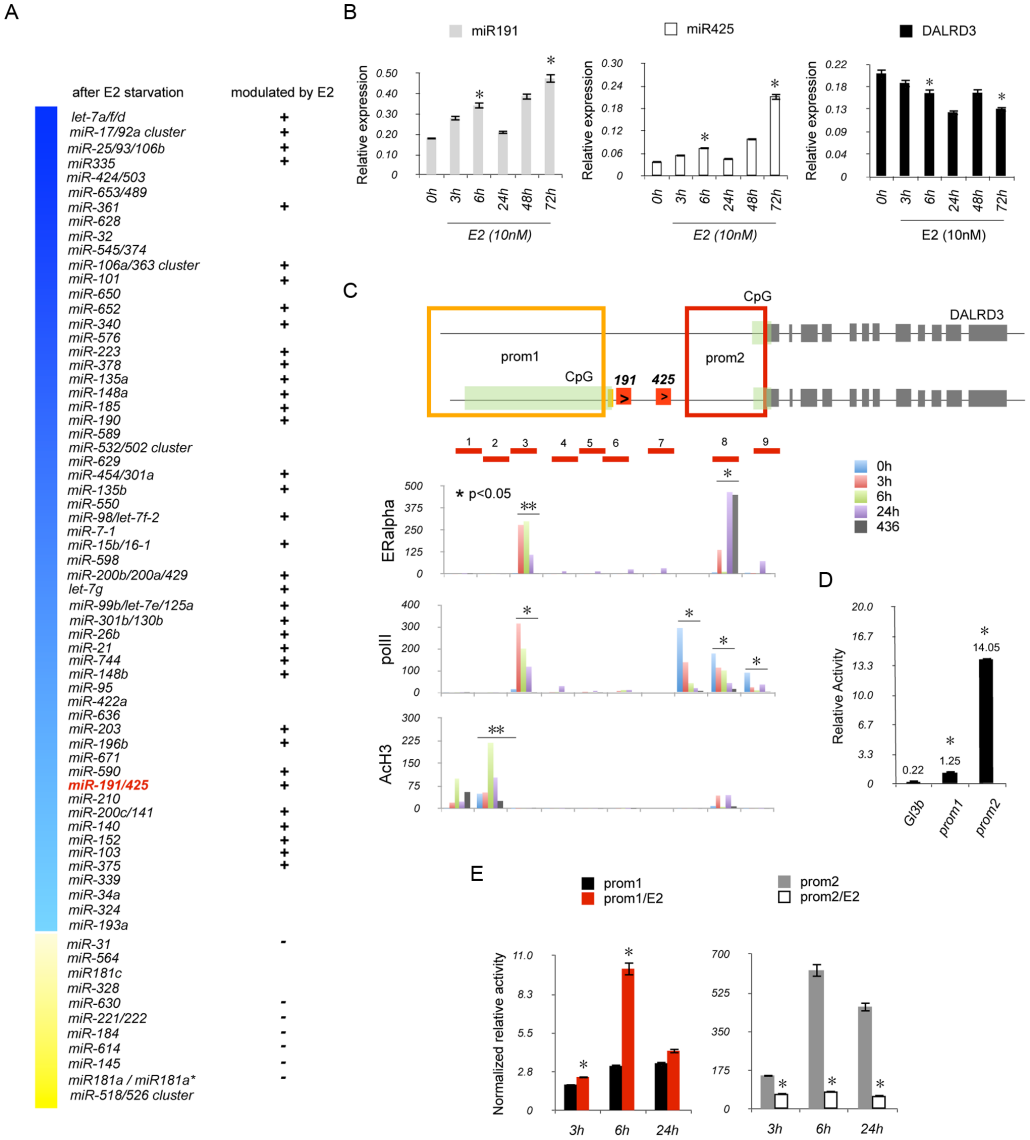
ERα regulates miR-191/425 cluster. (A) Schematic representation of E2 modulated miRNA genes (p-value<0.05 and fold change >1.2) after 6 days of hormone starvation and 6 h of E2 treatment. Blue (down) and yellow (up) columns represent all miRNA genes regulated after hormone starvation. (B) Mature miR-191, miR-425 and DALRD3 mRNA levels were determined by qRT-PCR (* p-value<0.05) after E2 treatment (10 nM). (C) Schematic representation of miR-191/425-DALRD3 transcription unit. Green, red and gray boxes depict CpG islands, miR-191/miR-425, and DALRD3 exons, respectively. Prom1 and prom2 regions are represented as red and orange squares, respectively. The red lines across miR-191/425 locus define the location of the amplicons that were used for the chromatin immunoprecipitation experiments. For ChIP experiment, MCF7 cells were hormone starved for 6 days and then treated with E2 (10 nM) for 3 h, 6 h and 24 h. The amount of ChIP-enriched DNA was quantified by quantitative PCR (qPCR), and the results are shown as relative enrichment to the input. (D) Luciferase assays were performed on prom1 and prom2 in HEK293 cells 24 h after transfection (* p-value<0.001). (E) Luciferase assays for prom1 and prom2 after estrogen stimulation. MCF7 cells were hormone starved for 4 days and then transfected with prom1 and prom2 plasmids. 24 hr post-transfection, cells were treated with E2 (10 nM) and luciferase activity assessed at the reported time points (* p-value<0.05). Error bars indicate s.d. and p-values were obtained with two-sided Student's t-test.

Recently, various microarray approaches have been used to identify E2-induced miRNA expression in hormone-dependent breast cancer cells [17]–[20]. However, based on the lack of consensus on E2-regulated changes in miRNA expression [30], we investigated global changes in endogenous miRNA expression after E2 stimulation of breast cancer cells using the multiplexed Taqman microRNAs assay, a highly sensitive technology that allowed us to detect changes in 754 miRNAs (“miRNome”) with the same sensitivity of a Taqman realtime PCR. ERα positive MCF7 cells were hormone starved for 6 days and then exposed to 10 nM of E2 for 6 h. The miRNome was determined at 2, 4, 6 days of hormone deprivation and 6 h after E2 stimulation (Figure 2A and Table S1). After 6 days of E2 deprivation, downregulation of 146 and upregulation of 25 mature miRNAs, organized in 69 different miRNA genes, were observed (fold change 1.2, p-value<0.05) (Figure 2A). Of these 69 miRNA genes, 43 genes (85 mature miRNAs) were modulated after 6 h of E2 stimulation (Figure 2A). The miR-191/425 cluster showed a progressive downregulation during the 6 days of hormone deprivation (p-value<0.05; fold change miR-191: 2 d: 0.82; 4 d: 0.63; 6 d: 0.45; miR-425: 2 d: 0.81; 4 d: 0.78; 6 d: 0.35) followed by a significant induction by 6 h of E2 stimulation (p-value<0.05; fold change miR-191: 1.36; miR-425: 1.12) (Table S1). We assessed the reliability of the treatment by using qRT-PCR to evaluate the expression levels of the E2-regulated genes, TFF1/pS2 and miR-17 after 3, 6, 24, 48 and 72 h of E2 stimulation [19] (Figure S5A). Both genes showed a strong and stable induction over time after E2 treatment. Next, we performed qRT-PCR on miR-191 and miR-425 and both miRNA levels increased after E2 stimulation although with a different kinetic of induction compared to miR-17 (Figure 2B). Specifically, after 72 h of E2 treatment, we detected a 2- to 3.5-fold induction of miR-191 and -425 compared to untreated cells and the presence of a block in their induction at 24 h after E2 treatment (Figure 2B). Next, we assessed expression levels of the primary precursor of miR-191 and miR-425; the induction profile was similar to the mature miRNAs (Figure S5B). Despite the positive correlation between miR-191/425 and the host gene DALRD3 in breast cancer cells (Figure 1C, 1D), the expression level of the total DALRD3 mRNA was decreased of 35% after 72 h of E2 treatment compared to untreated cells (p-value = 0.053) (Figure 2B). qRT-PCR for the two different alternative splicing variants of DALRD3 also showed a repression of both variants after estrogen stimulation (Figure S5C). Moreover, total DALRD3 mRNA and both variants were also highly upregulated in hormone-deprived MCF7 cells (Figure S5D). To further confirm the ability of E2 to modulate miR-191/425, MCF7 were treated with fulvestrant, an ERα antagonist that induces ERα protein degradation (Figure S6A). We observed a consistent reduction in miR-191/425 levels and a constant increase in DALRD3 levels after fulvestrant treatment (Figure S6B, S6C). TFF1/pS2 expression was downregulated by hormone deprivation or fulvestrant treatment (Figure S5D; Figure S6C). Collectively, the data showed that miR-191/425 levels are positively regulated by ERα, and the increased levels of miR-191 and miR-425 after estrogen stimulation are associated with a reduction in the accumulation of the host gene DALRD3.

### ERα directly regulates miR191/425 cluster

Next, we addressed the direct involvement of ERα in the regulation of miR191/425 cluster by performing chromatin immunoprecipitation (ChIP) experiments across nine different regions spanning miR-191/425 cluster and covering a region of 4200 bp (Figure 2C). MCF7 cells were E2 starved for 6 days (0 h) and then treated with E2 (10 nM) for 3 h, 6 h and 24 h. Enrichment of ERα after E2 treatment was identified at region 3 and 8 (Figure 2C). Region 3 showed a specific enrichment of ERα that reached the highest levels after 3–6 h of treatment and started to decrease at 24 h. Although ERα was also detected at region 8 after 3 h and 24 h of E2 treatment, this enrichment was considered to be non-specific since it was also detected for the ERα negative MDA-MB-436 cells (Figure 2C). We also examined the localization of the non-phosphorylated RNA polymerase II large subunit (polII) and the acetylation status of the histone H3 (AcH3) after E2 treatment (Figure 2C). Immunoprecipitation against polII showed the presence of two different areas of enrichment: region 3, with an E2-dependent recruitment of polII that decreased over time, and region 7–9 which showed a progressive reduction in polII recruitment during E2 treatment (Figure 2C). AcH3 ChIP showed a specific enrichment at region 1, 2 and 8 with a significant increase in H3 acetylation after 6 h of E2 treatment only for region 2 (Figure 2C). Taken together, these experiments show that ERα is recruited to the miR-191/425 genomic locus, in response to the estrogen stimulation.

Because of the presence of two sites of enrichment of polII and the presence of two CpG islands located at the 5′end of the two isoforms of DALRD3 (Figure 2C), we hypothesized the existence of two promoter regions: one responsible for the transcription of the longest isoform of DALRD3, which includes miR-191 and -425 and a second responsible only for the transcription of the short isoform of DALRD3. Computer-assisted analysis (Figure S7A) identified two distinct predicted regions as possible candidates for promoters regulating miR-191/425/DALRD3 gene transcription: 3900 bp (prom1) a marginal predicted region, located upstream of the long isoform of DALRD3 and also involved in the production of miR-191/425; 6500 bp (prom2) a highly likely predicted region, associated only to the transcription of the short isoform of DALRD3 mRNA (Figure S7A). To test the transcriptional activity of these two elements, both putative promoters (Figure 2D) were cloned individually in the promoter-less pGL3basic luciferase vector, and their expression was examined in HEK293 cells. Both vectors showed an increase in the luciferase activity, and as expected, the highly likely predicted region prom2 showed the strongest basal luciferase activity (Figure 2D). Next, we assessed the E2 responsiveness of the two identified promoter regions. We first tested the luciferase activity of both plasmids in five breast cancer cell lines with different ERα expression levels (Figure S7B). Both promoter elements showed higher levels of activity in the three ERα positive cell lines (MCF7, T47D, BT-474) compared to the ERα negative cells (BT-459, MDA-MB-436). Treatment with E2 for 6 h induced a 3-fold increase in luciferase activity for the prom1 element (Figure 2E); in contrast, luciferase activity for the prom2 region was repressed by E2 treatment (Figure 2E). Furthermore, silencing of ERα by siRNA reduced luciferase activity of the prom1 reporter vector by approximately 50% specifically in ERα positive cells, but no effect on prom2 activity was detected (Figure S7C).

Taken together, these experiments showed that (1) ERα directly regulated miR-191/425 cluster expression and (2) verified the existence of two promoter elements involved in the transcription of the two DALRD3 isoforms, allowing a differential accumulation of miR-191/425 and DALRD3 upon E2 stimulation.

### miR-191 controls EGR1 in ERα-positive breast cancer cells upon E2 stimulation

**Figure 3:**
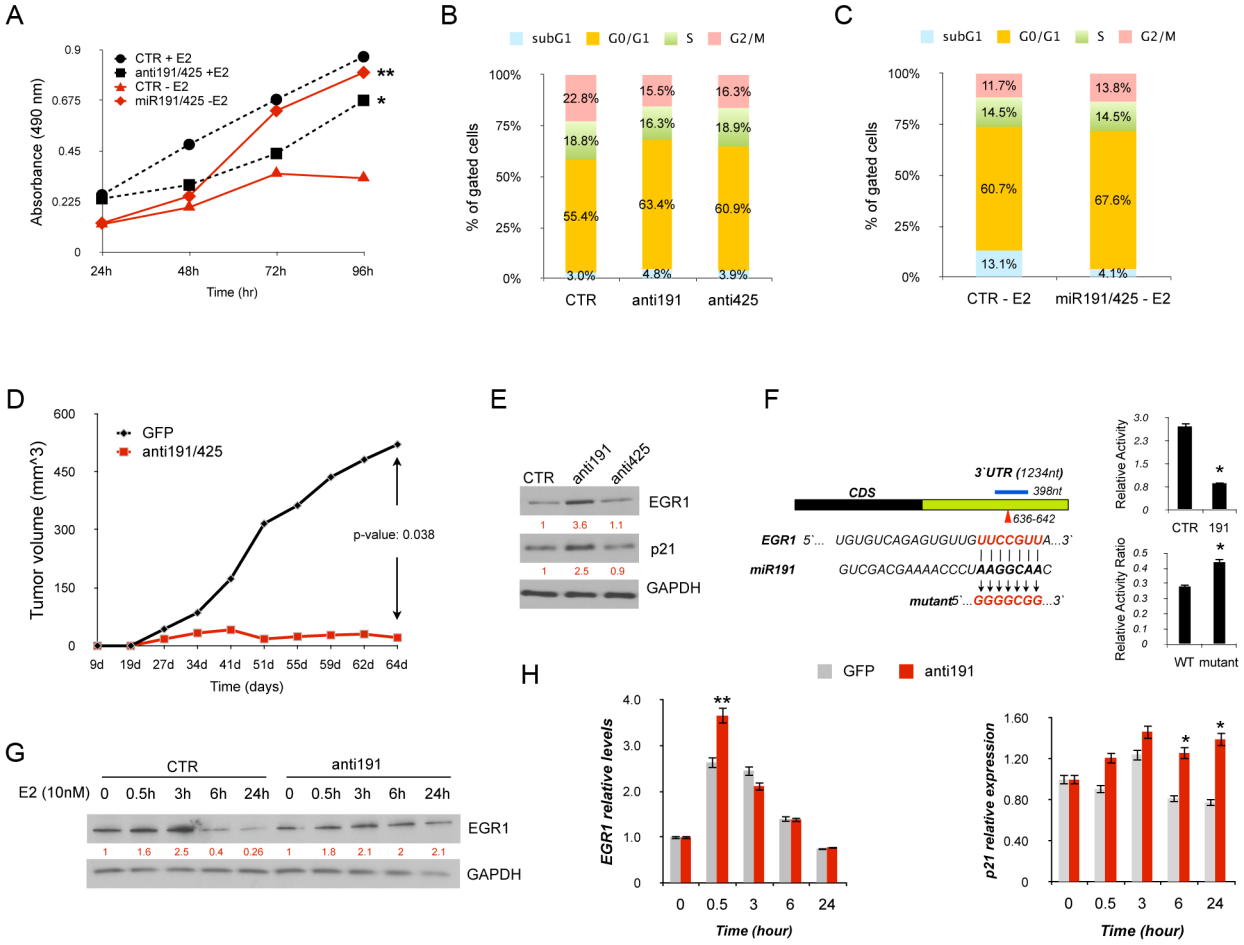
miR-191/425 cluster effects in ERα-positive breast cancer cells. (A) MTT assay in MCF7 cells transfected with anti-miR-191/425 and scrambled (CTR) control oligonucleotide in normal culture condition (+E2) and MCF7 cells transfected with miR-191/425 and scrambled (CTR) control in hormone starvation for 7days (−E2) (* and ** indicate p-value<0.05 and 0.01, respectively). (B) Cell cycle analyses of MCF7 cells transfected with anti miR-191/425 and scrambled control (CTR) oligonucleotide in normal culture condition. Cells were harvested 72 h following transfection, fixed, stained with propidium iodide, and analyzed by flow cytometry; the data are representative of three independent experiments. (C) Cell cycle analyses of MCF7 cells transfected with miR-191/425 and scrambled control (CTR) oligonucleotide. Briefly, MCF7 cells were hormone deprived for 3 days and then transfected with miRs and control oligonucleotide. Cells were harvested 48 h following transfection, fixed, stained with propidium iodide, and analyzed by flow cytometry; the data are representative of three independent experiments. (D) *In vivo* growth kinetic of MCF7 cells transfected with anti-miR-191/425 and scrambled control oligonucleotide. Briefly, MCF7 were transfected in 10 cm plates by using 2-O-methyl anti miR-191 and miR-425 oligonucleotides (100 nM); 48 h after transfection, cells were detached and injected in nude mice previously implanted (two weeks before injection) with estradiol pellets. p-value was calculated on one experiment performed with 6 mice for each group. (E) Levels of EGR1 and p21 were measured by western blot analyses 72 h after transfection of anti-miR-191, anti-miR-425 and scrambled oligonucleotide control in MCF7 cells. Densitometric values are reported in red below each band and represent the average of three independent experiments. GAPDH was used as a loading control. (F) Schematic representation of miR191 binding site located in the 3′UTR (in green) of the target gene EGR1. The blue line defines the 3′UTR fragment cloned into pGL3 control plasmid and the arrow head indicates the position of the miRNA binding site, whose sequence is reported below with the mutagenesis strategy used to generate the disruption between miRNA and mRNA of the target gene. Luciferase assays for wildtype and disrupted miRNA binding sites: (top panel) reduced luciferase activity after miR-191 overexpression; (lower panel) increased luciferase activity after miR191 enforced expression on mutated plasmid. In the lower panel, luciferase activity values for wildtype and mutated plasmids are represented as a ratio between the relative luciferase activity of miR-transfected cells with the relative luciferase activity of control-transfected cells. * represent p-value<0.01. (G) Western blot for EGR1 protein after estrogen treatment of miR-191 knocked-down MCF7 cells. Briefly, MCF7 cells were hormone deprived for 2 days and then transfected by using 2-O-methyl anti miR-191 (100 nM); 48 h after transfection cell were stimulated with E2 (10 nM) and collected at the reported time points. Densitometric values are reported in red below each band and represent the average of three independent experiments. GAPDH was used as a loading control. (H) qRT-PCR for EGR1 and p21 mRNAs after miR-191 inhibition in E2 stimulated MCF7 cells. Gene expression levels are reported as relative expression to GAPDH levels. Error bars indicate s.d. *, ** represent p-value of 0.05 and 0.01, respectively.

To identify the functional role of the E2 mediated-induction of miR-191 and miR-425 in ERα positive breast cancer cells, both miRNAs were knocked down in estrogen dependent MCF7 cells in normal culture condition. A 33% reduction in cell proliferation rate was observed compared to a control oligonucleotide (Figure 3A). Indeed, enforced expression of miR-191/-425 in hormone deprived MCF7 cells, with low levels of endogenous miR-191/425 (Table S1), induced a 70% increase in cell proliferation (Figure 3A). To shed more light in the proliferative effects of miR-191/425 in ERα positive breast cancer cells, flow cytometric analyses of transiently-transfected cells were performed and revealed an increased number of cells in G_1_ and fewer cells in G_2_/M following knockdown of either miR-191 or miR-425 compared to control cells (Figure 3B and Figure S8A). Moreover, enforced expression of miR-191/425 in hormone deprived MCF7 cells protects cells from hormone starvation induced apoptosis (Figure 3C).

We next evaluated the *in vivo* effect of miR191/425 knockdown on tumor growth. Specifically, miR-191/425 were transiently inhibited in ERα positive MCF7 cells for 48 h and tumor growth was assessed after subcutaneous transplantation of the transfected MCF7 cells in nude mouse. A 50% reduction in tumor growth was observed (Figure 3D) in miR-191/425 knocked-down cells compared to control cells. Same results were also obtained after xenotrasplantation of miR-191/425 knocked-down ERα positive ZR-75-1 cells (Figure S8B).

To uncover the molecular players involved in the proliferative response of ERα positive breast cancer cells controlled by the E2 mediated activation of miR-191/425, published transcriptomic data set of E2 induced ERα positive MCF7 and ZR-75-1 cells were compared with the predicted miR-191/425 target genes [31], [32]. Specifically, the target genes of miR-191 and miR-425 obtained from the prediction program Targetscan v5.2 (Table S2) were compared with the pool of E2 downregulated genes. 43 and 23 miR-191 targets and 199 and 116 miR-425 targets were found in the E2 repressed gene lists of MCF7 and ZR-75-1, respectively (Figure S9A). Only 5 and 18 targets for miR-191 and miR-425 were repressed by estrogen in both cell lines respectively (Figure S9A). We focus our attention on the early growth response 1 (EGR1), a member of the early growth response (EGR) transcription factor family that has been implicated in breast cancer progression and antiestrogen resistance [33]–[35]. First, the expression levels of EGR1 were assessed after E2 stimulation in MCF7 cells. EGR1 expression showed a 50% induction after 30 minutes from the stimulation (Figure S9A) followed by a continuous repression (Figure S9B). To verify that miR-191 regulates the expression of EGR1, knockdown of miR-191 was performed in MCF7 cells and western blot analyses confirmed the upmodulation of EGR1 and its direct transcriptional target CDKN1A (p21) (Figure 3E) [34]. Next, to assess that miR-191 directly controls EGR1 in cells, a luciferase reporter assay was performed with a luciferase expressing plasmid containing the conserved miR-191 predicted binding site for EGR1 cloned after the luciferase reporter gene (Figure 3F). Co-transfection of miR-191 with the reporter plasmid significantly suppressed (p-value<0.01) the luciferase activity of the reporter, relative to transfection of the control oligonucleotide (Figure 3F). Disruption of the predicted binding site reduced the inhibitory activity of miR-191 overexpression on the luciferase activity (Figure 3F). To study in more depth the interaction miR-191/EGR1, hormone deprived MCF7 cells were transfected with miR-191 inhibitor and control oligonucleotide and 48 h later treated with estradiol. Western blot analyses showed that miR-191 inhibition prevents EGR1 degradation at 6 h and 24 h after E2 treatment compared to control cells (Figure 3G). qRT-PCR showed that EGR1 mRNA is also under the control of miR-191 but only in the early phase of E2 induction (Figure 3H). As expected, induction of p21 transcript was confirmed by qRT-PCR specifically in miR-191 knocked-down (Figure 3H). MiR-191 inhibition was also confirmed by qRT-PCR (Figure S9C). Taken together, these results highlight the proliferative effects of E2-induced miR-191/425 cluster in ERα positive breast cancer cells that are in part related to the miR-191 repression of the tumor-suppressor gene EGR1.

### miR191/425 cluster modifies gene expression in highly aggressive breast cancer cells

**Figure 4:**
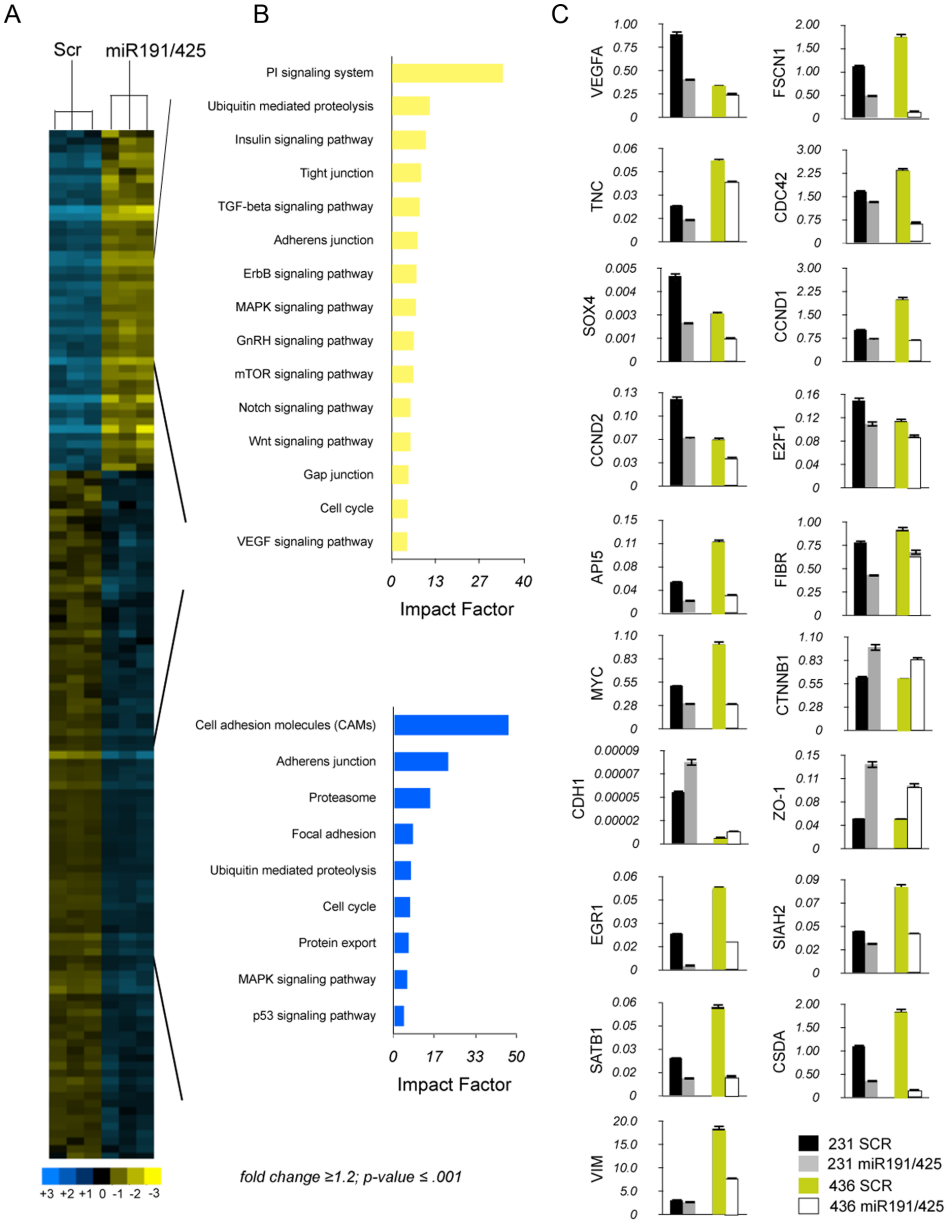
miR-191/425 cluster alters gene expression profile of highly aggressive MDA-MB-231. (A, B) Unsupervised clustering of genes differentially expressed between miR-191/425 and scrambled oligonucleotide control transfected MDA-MB-231 cells. (C) qRT-PCR for the miR-191 and miR-425 modulated genes in two different aggressive breast cancer cells after miR-191/425 over-expression. Gene expression levels are reported as relative expression to GAPDH levels. All graphs showed p-values<0.05 obtained with two-sided Student's t-test. Error bars indicate s.d.

Approximately 75% of diagnosed breast tumors express ERα, and this ERα-positive status is associated with a better prognosis and response to hormonal treatment [36]. Several studies suggested that a fraction of ER-negative tumors arise from ER-positive precursors [37]. Moreover, restoration of functional ERα expression in ERα-negative human breast cancer cells can block their proliferation and aggressiveness, supporting the notion that ERα confers a less aggressive phenotype of breast cancer [38], . To determine if miR-191/425 cluster as a part of the ERα signaling can partially mediate the anti-proliferative effect that ERα showed in the aggressive breast cancer cells, a genome-wide expression analysis in aggressive MDA-MB-231 cells, which express low levels of miR-191/425, was performed 72 h after enforcing expression of both miR-191 and miR-425 and control oligonucleotide (Figure 4A). Unsupervised clustering analyses showed significant deregulation of gene expression by miR-191/425, with 753 upregulated and 1105 downmodulated genes (by>1.5 fold; p-value 0.001) (Table S2). Functional profiling of these genes defined that the greatest proportion of them is associated with cell adhesion, adherens junction followed by phosphatidylinositol signaling (Figure 4B). We used qRT-PCR to validate the modulation of over 20 genes identified in the microarray analyses or to their related molecular pathways in two different breast cancer cell lines (Figure 4C). Expression of many genes involved in promoting growth and metastasis of breast cancer cells was found to be downmodulated by miR-191/425 cluster: CCND1, CCND2, E2F1, CSDA and API5, regulatory proteins of the cell cycle progression and apoptosis [40]–[42]; FSCN1, TNC, VEGFA, CDC42 and SOX4, which have roles in angiogenesis and migration, and are involved in filopodia/invadopodia formation [43]–[48]; the protooncogene MYC, which initiates the transcription of a large set of genes involved in cell growth by stimulating metabolism and protein synthesis [49]; and SATB1, which reprograms gene expression to enhance aggressive histomorphological features and invasive capabilities [50]. We also found that miR-191/425 cluster represses cell-structure and adhesion genes typical of invasive breast cancer cells such as fibronectin, an ECM adhesive glycoprotein, and vimentin, the intermediate filament protein of mesenchymal cells, which together provide cellular integrity and resistance against stress [51]. Finally, miR-191/425 cluster upregulates zonula occludens-1 (ZO-1), a component of the tight junction barrier in epithelial and endothelial cells [52]; E-cadherin (CDH1), an important marker of epithelial tumor progression; and β-catenin (CTNN1) a component of *wnt* pathway that drives progression in various cancers [53].

**Figure 5:**
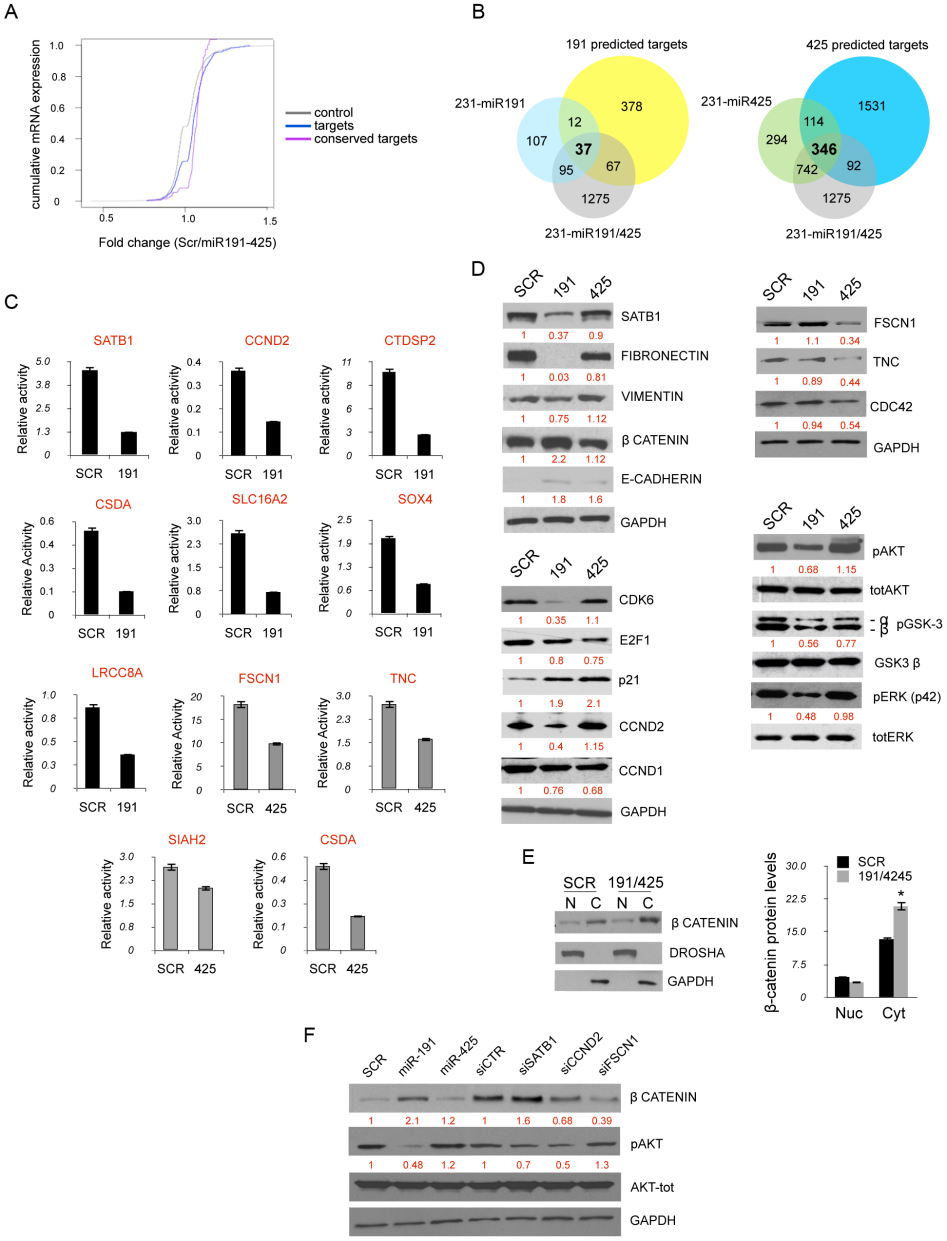
miR191/425 targets identification. (A) Cumulative distribution functions (cdfs) of log_2_ fold change of mRNA expression between miR-191/425 and scrambled control cells is plotted. Plots include conserved (pink), predicted miR-191/425 targets (blue line), and control mRNAs (grey line). Targets include ∼2600 predicted TargetScan v5.1 targets of miR-191 and miR-425. Conserved targets contain the ∼150 genes ranked by TargetScan v5.1 branch length scores. The control mRNA set was selected to match the predicted targets in expression, 3′UTR length and composition. p-value ″ 2.2e^−16^ by rank sum test. (B) Intersection of predicted miR-191, miR-425 human targets and miR-191/425, miR-191, miR-425 repressed genes. (C) Luciferase activity was measured 24 h after transfection of HEK293 cells with reporter plasmids in which miR-binding sites listed in Figure S8 were fused downstream the firefly luciferase gene. Reporter plasmids were transfected either with miR-191 or miR-425 and miR-negative control (SCR). All graphs showed p-values<0.001 obtained with two-sided Student's t-test. (D) miR-191/425 modulated proteins were analyzed by western blot analyses 72 h after miR-191 and miR-425 over-expression in MDA-MB-231. (E) Increased of β-catenin protein levels in the cytoplasmic fraction of miR-191/425 transfected MDA-MB-231. Immunoblots show the protein level of β-catenin in the nuclear and cytoplasmic fraction, whereas DROSHA and GAPDH were used as loading controls for the nuclear and cytoplasmic proteins, respectively. Densitometric analyses of β-catenin protein levels are shown. * represent p-value<0.01. (F) Modulation of pAKT, β-catenin after SATB1, CCND2, FSCN1 silencing. Vinculin is used as a loading control. All error bars indicate s.d.

To confirm that targets of the mir-191/425 cluster showed an enrichment signature in this dataset, we assessed the cumulative density function (cdf) plot comparing the expression changes of mir-191 and miR-425 targets based on TargetScan v5.1 gene list [54]. We found that the mir-191/425 targets set (targets) was more repressed than the control set of genes (control) matched for 3′UTR length, dinucleotide composition, and expression level (Figure 5A). Stronger repression was observed for the conserved miR-191/425 cluster targets (conserved targets), suggesting further enrichment of genuine targets in this set (Figure 5A). These observations supported the utility of this expression data for the discovery of novel miRNA targets based on miR-associated genes. Because the expression levels of target mRNAs tend to correlate negatively with the expression levels of their specific miRNAs [55], we next focused on the miR-191/425 downregulated genes. First, the target prediction program TargetScanv5.1 was used to search for predicted target genes of miR-191 and miR-425 in the pool of downregulated genes in miR-191/425-expressing MDA-MB-231 cells (Table S2). This list of genes was further compared with the list of target genes downregulated exclusively by the expression of miR-191 or miR-425 (Figure S10 and Table S2). A total of 37 and 346 downregulated targets were obtained for miR-191 and miR-425, respectively (Figure 5B). Among these large set of genes, we selected 12 genes (SATB1, CCND2, CTDSP2, SOX4, LRRC8A, SLC16A2, CSDA for miR-191 and FSCN1, TNC, SIAH2, CCND1, CSDA for miR-425) predicted to have at least one potential binding site for miR-191 and/or mir-425 in their 3′UTRs. Based on their reduction in miR-191/425-expressing cells (Figure 4C and Table S2), we tested whether these genes are direct targets of miR-191 and miR-425 constructing reporter plasmids containing the miRNA binding site in the 3′UTR of these genes downstream of a *luciferase* reporter gene (Figure S11A). Co-transfection experiments showed that the introduction of either miR-191 or miR-425 markedly suppressed the expression of a *luciferase* containing the 3′UTR of these downregulated genes (Figure 5C) but did not affect the luciferase activity of the 3′UTR-CCND1 plasmid, indicating that CCND1 is not a direct target of miR-425 (data not show). Mutations that disrupt base paring with miR-191 and miR-425 rescued the luciferase expression for all the target genes, further confirming that these genes are direct targets of miR-191 and miR-425 (Figure S11B).

We next focused our attention exclusively on SATB1, CCND2 and FSCN1 as mediators of miR-191 and miR-425 effects, respectively, because of their strong repression obtained after miRNA expression and their reported tumorigenic function in breast cancer [46], [50], [56], [57]. Western blot analyses on MDA-MB-231 expressing either miR-191 or miR-425 showed a strong suppression of SATB1 only after enforced miR-191 expression (Figure 5D). Because of SATB1 repression, we also detected marked repression of fibronectin and to lesser extent of vimentin (Figure 5D). Further, we also observed a ∼2 fold increase of the β-catenin protein (Figure 5D) and its sequestration at the cytoplasmic membrane due to the increased expression of e-cadherin (Figure 5D, 5E). Indeed, miR-191 over-expressing cells also showed a specific repression of CCND2 as well as CDK6 (Figure 5D), a previously demonstrated miR-191 target [28]. Furthermore, we observed a decrease in the levels of CCND1, E2F1 and a strong upmodulation of CDKN1A (p21) for both miR-191 and miR-425 (Figure 5D). In contrast, miR-425 over-expression specifically reduced expression of FSCN1, TNC and CDC42 (Figure 5D). Pathway analyses also revealed a repression of the PI3K-AKT pathway in miR-191/425 over-expressing cells. Western blot analyses against pERK1/2, pAKT and its direct targets pGSK3β confirmed the inhibition of PI3K-AKT signaling and highlighted that miR-191 is primarily responsible for the inhibition (Figure 5D). Moreover, we performed silencing of SATB1, CCND2 and FSCN1 in order to evaluate the specific contribution of each target to modulated miR-191/425 pathways. We found that only SATB1 knockdown, as well as miR-191 over-expression, were responsible for the up-modulation of β-catenin, whereas both CCND2 and FSCN1 silencing decreased β-catenin expression (Figure 5F). Finally, we found that SATB1 and CCND2 silencing controlled AKT pathway activation (Figure 5F). Taken together, these data indicate that miR-191/425 modify a number of genes that play critical roles in controlling the progression of highly invasive breast cancer.

### miR191/425 cluster impairs tumorigenicity and aggressiveness of breast cancer cells

**Figure 6:**
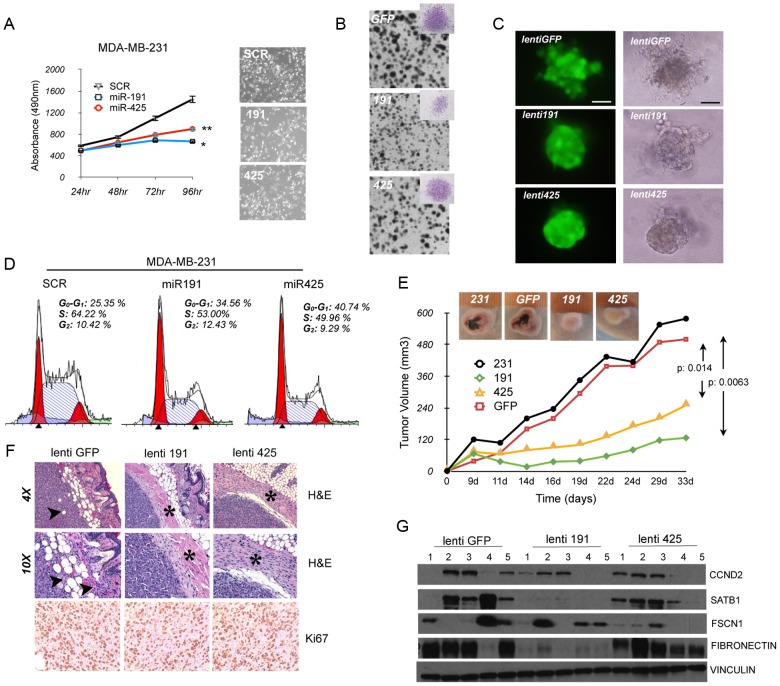
miR-191/425 cluster impairs tumorigenicity of aggressive breast cancer cells. (A) MTT assay in miR-191, miR-425 and scrambled (SCR) control transfected MDA-MB-231 cells (* and ** indicate p-value<0.05 and 0.01, respectively). Microscopic photos of representative miR transfected MDA-MB-231 cells (4× magnification). (B) 2D colony formation assay in miR-191, miR-425, or empty vector control (GFP) lentivirally-infected MDA-MB-231 cells. Representative micrograph of crystal violet-stained colonies. (C) Fluorescent (left) and bright field (right) images of miR-191, miR-425, or empty vector control (GFP) lentivirally-infected MDA-MB-231 cells grown in Matrigel for 5 days. Bars, 100 µm. (D) Cell cycle analyses of miR-191, miR-425 and scrambled control (SCR) transfected MDA-MB-231 cells. Cells were harvested 72 h following transfection, fixed, stained with propidium iodide, and analyzed by flow cytometry; the data are representative of three independent experiments. (E) *In vivo* growth kinetics of parental MDA-MB-231 cells compared to miR-191, miR-425, or empty vector control (GFP) lentivirally-infected cells. Images show average-sized tumors for each group. The p-values were calculated on two different experiments performed with 5 mice per group. (F) Hematoxylin-eosin (H&E, upper and middle rows) and Ki67 staining of xenograft tumors at 7 weeks after subcutaneous transplantation of miR-191, miR-425, or empty vector control (GFP) lentivirally infected-MDA-MB-231 cells. Arrows in panels indicate areas of fat invasion, while asterisks identify the fibrous capsule. (G) Levels of CCND2, SATB1, FSCN1 and fibronectin were measured by western blot analyses in 5 tumors of each experimental group. Vinculin was used as a loading control.

Next, we assessed the *in vitro* biological effect of miR-191/425 on aggressive breast cancer cells. First, enforced expression of miR-191 or miR-425 in MDA-MB-231 and MDA-MB-436 cells induced an approximately 50% reduction in cell proliferation (Figure 6A and Figure S12A). Lentivirally-infected cells over-expressing either miR-191 or miR-425 were generated (Figure S12B), and cell proliferation was assessed using a (2D) colony formation assay (Figure 6B and Figure S12C). Cells over-expressing miR-191 not only showed a reduced number of colonies compared to control but also developed smaller colonies than control (Figure 6B and Figure S12D); in contrast, miR-425-expressing cells exhibited mainly a reduction in the number of colonies (Figure 6B and Figure S12D). Further, we tested the abilities of lentivirally-infected MDA-MB-231 cells to form colonies in soft agar. Compared to control cells, cells over-expressing either miR-191 or miR-425 formed significantly fewer colonies, indicating a decrease in anchorage-independent growth (Figure S12E). We then performed proliferation assays with cells cultured in three dimensions (3D) within Matrigel, and we observed that over-expression of either miR-191 or miR-425 impaired the formation of large filopodia/invadopodia-like structures at the periphery of the aggregates like in the control cells, thus resulting in the appearance of tightly adherent aggregates (Figure 6C). These results demonstrated that gain of cell adhesion and reduced migration are related to the degree of miR-191 and miR-425 expression in aggressive breast cancer cells. To more accurately quantify the anti-proliferative properties of miR-191/425 in aggressive breast cancer cells, flow cytometric analyses of transiently-transfected cells revealed fewer cells in S phase and an increased number of cells in G_1_ following over-expression of either miR-191 or miR-425 compared to scrambled transfected cells (Figure 6D and Figure S12F). To gain additional insight regarding the numbers of cells arrested in G_1_, we treated the cells with the microtubule-destabilizing agent nocodazole, which traps cycling cells in M phase. Cell populations with enforced miR-191 or miR-425 expression were characterized by significantly increased numbers of cells remaining in G_1_ (Figure S12G), confirming that both miRNAs caused cell-cycle arrest.

We next evaluated the *in vivo* effect of miR191/425 over-expression on tumor growth. First, we tested if over-expression of either miR-191 or miR-425 inhibits tumor growth of highly aggressive MDA-MB-231 cells. Lenti-miR-191 and lenti-miR-425 infected MDA-MB-231 were subcutaneously injected into the right flank of athymic nude mice and the tumor growth was monitored compared to control lenti-GFP infected and parental MDA-MB-231 cells. Tumors in the parental and GFP control groups were large, poorly differentiated, heavily necrotic and highly vascularized that formed within only 22 days post-implantation (5 out of 5 mice per group). In contrast, all five mice injected with either miR-191- or miR-425-infected cells exhibited greatly reduced tumor growth (Figure 6E). Interestingly, miR-191 and miR-425 over-expressing tumors were strictly non-invasive, as shown by their circumscribed profiles and confinement within dense fibrotic capsules (Figure 6F), in stark contrast to the spindle-like morphology of the parental and control tumors along with islands of cancer cells invading the fat pad and the muscle (Figure 6F and Figure S13A). Hence, ectopic expression of miR-191 and miR-425 in MDA-MB-231 cells impaired tumor growth and invasion in the surrounding tissue. To determine whether miR-191 and miR-425 expression in the primary tumors affects cell proliferation, we performed immunohistochemistry for the proliferation marker Ki-67. We found that the total number of Ki-67 positive cells in the tumors over-expressing miR-191 or miR-425 were significantly lower relative to the number observed in the control tumors (lenti-GFP control cells: 97.3%; lenti-miR-191: 81%; lenti-miR-425: 89%; p-value<0.05) (Figure 6F). High expression of miR-191 and miR-425 in the tumor cells was confirmed by qRT-PCR (Figure S13B). qRT-PCR revealed that miR-191 induced a reduction of mesenchymal (fibronectin) and acquisition of epithelial (e-cadherin and β-catenin) markers while miR-425 only a specific increase in e-cadherin (Figure S13C). Reduction of SATB1, CCND2 by miR-191 and FSCN1 by miR-425 over-expressing tumors was confirmed by western blot analyses (Figure 6G; and Figure S13D). Based on these numerous observations, we concluded that the impaired tumor growth of miR-191- or miR-425-over-expressing cells was a consequence of the reduced cell proliferation.

**Figure 7:**
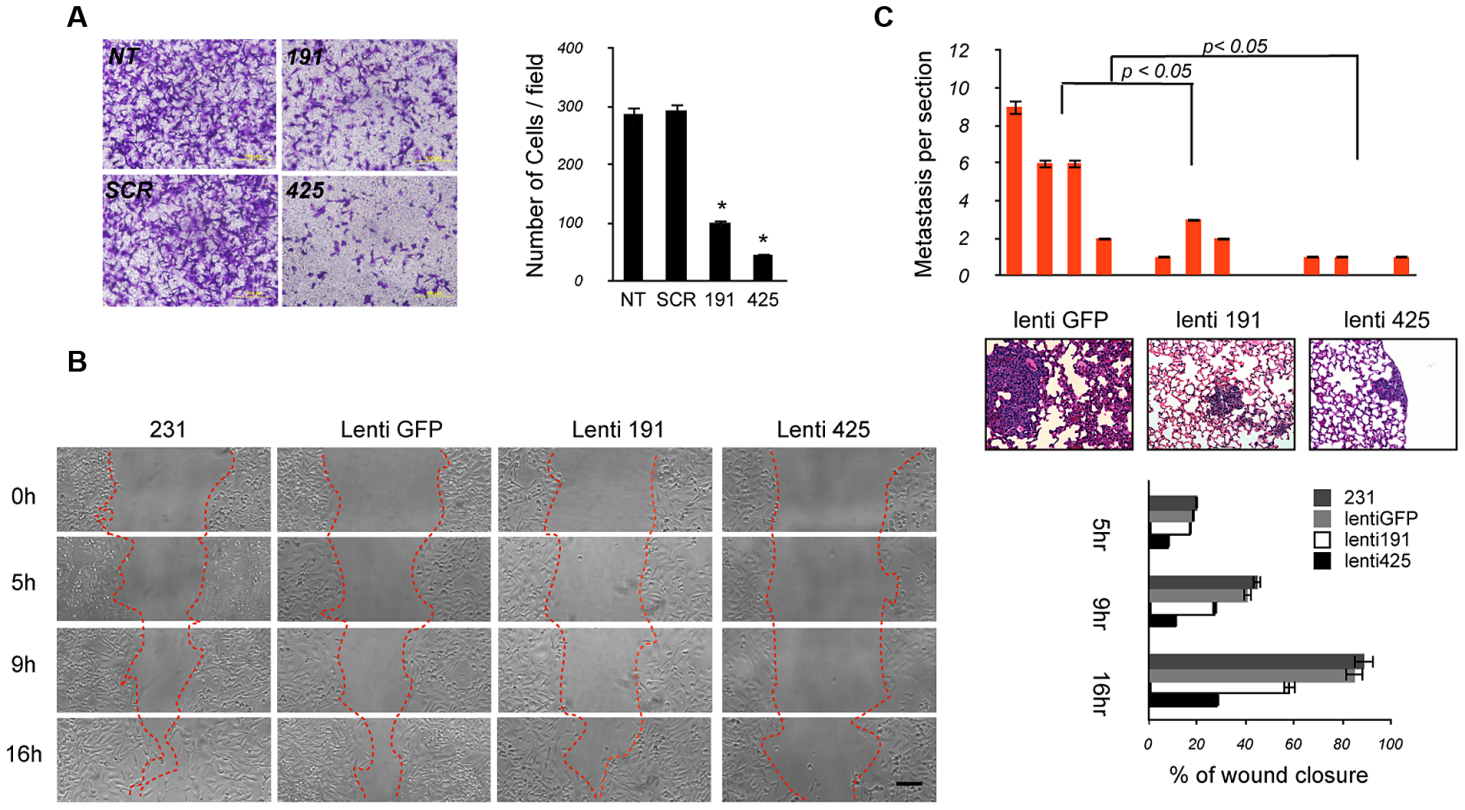
The miR-191/425 cluster reduces migration and metastatic dissemination of breast cancer cells. (A) Representative pictures taken at 10× magnification of crystal-violet-stained parental (NT), miR-191, miR-425 and scrambled control transfected MDA-MB-231 cells migrated through inserts 24 hrs after plating. Collected data from three separate experiments performed in triplicate are represented as number of cells per field. Error bars indicate s.d. And * represent p-value<0.05. (B) *In vitro* wound-healing assay comparing the progressive extent of migration by miR-191, miR-425, or empty vector control (GFP) lentivirally-infected MDA-MB-231 cells. The photomicrographs were taken at 10× magnification; bar, 100 µm. The widths of wounds were measured on the microscopic photos at 0, 5 h, 9 h and 16 h after wounding. Changes in wound width were converted to percentages to represent wound closure. Error bars indicate s.d. and p-values<0.05. (C) Lung colonization assays of MDA-MB-231 cells after injection into the tail vein of NOD-SCID mice (4 mice per cell line, 2×10^6^ cells per mouse). Eight weeks after injection, lungs were analyzed for the presence of micrometastatic nodules. Analyses were carried out on H&E-stained histological sections (5 sections per lung). Data are represented as mean (bars) and s.d. (p-values<0.05). Representative pictures of metastases embedded in the lung parenchyma.

We then assessed the effects of miR-191/425 over-expression on migration and metastasis by using *in vitro* and *in vivo* experimental approaches. First, we evaluated the rate of cell migration by using the Boyden Chamber assay and found that miR-191- and miR-425-transfected cells migrated more slowly than control MDA-MB-231 cells (miR-191: p-value<0.05, ∼3-fold; miR-425: p-value<0.05, ∼6-fold) (Figure 7A). Further, we performed wound-healing assays on lenti-miR-191, lenti-miR-425 cells and GFP control (Figure 7B). By 16 hour post wounding, parental cells and GFP control cells migrated into the wound, resulting in 90% and 70% closure, respectively. In contrast, wound closure was significantly less in miR-191 and highly impaired in miR-425 (miR-191: 60% closed; miR-425: 25% closed) (Figure 7B). Migration and wound healing experiments were also performed using MDA-MB-436 cells, and the results were essentially similar (Figure S14A and S14B).

Finally, we tested the differential migratory abilities of miR-191 or 425-over-expressing cells by using an *in vivo* metastasis assay. Control lenti-GFP, lenti-miR-191, lenti-miR-425 infected-cells (2×10∧6 cells) were injected into the lateral tail vein of 6-week-old NOD-SCID mice, and their survival was evaluated in circulation, extravasation to and growth in lungs. After 8 weeks, histological analyses revealed that the number of micrometastasis was markedly reduced in the lungs of mice injected with miR-191 or miR-425 cells compared to the control tumor cells (Figure 7C). Of note, we also observed pneumonitis only in mice injected with the control GFP cells (Figure 7C). Collectively, all these data support the idea that sustained miR-191 and miR-425 activity impairs local invasion and metastatic colonization of breast cancer cells.

## Discussion

Defining the role of the differentially regulated miRNAs in breast cancer could lead to the development of new diagnostic tools and therapeutic approaches. In the present study, we provide new evidence for the role of miR-191 and miR-425 in breast cancer. We demonstrate that expression of miR-191 and miR-425 occurs as a part of the same transcriptional unit and strongly correlates with cellular ERα status. Moreover, we show that ERα directly regulates the expression of miR-191 and miR-425. Finally, our functional studies demonstrate that miR-191/425 cluster exerts a dual role in breast cancer cells depending on their ERα status: in ERα positive cells miR-191/425 work as oncogenes by inducing proliferation in part through the suppression of EGR1 during the E2 stimulation; in ERα negative cells, they impair tumor growth and invasiveness conferring a more epithelial phenotype to highly aggressive breast cancer cells.

We have demonstrated that miR-191 and miR-425 are co-expressed (Figure S4B) and, at least in part, transcriptionally dependent from the host gene DALRD3 in normal human tissues (Figure S2A, S2B). The identification of two distinct promoter regions responsible for the production of the two DALRD3 isoforms may allow the independent production of DALRD3 from the miRNAs and thus explain the partial correlation between miR-191/425 and DALRD3 found in some of the human tissues. Furthermore, the existence of the dual promoter for DALRD3 may contribute to “fine-tuning” of the estrogen-dependent regulation of miR-191/425 and DALRD3 gene transcription. We demonstrate that while E2/ERα signaling induces an increase in miR-191/425 expression ERα activation has a negative effect on the expression of the host gene DALRD3 (Figure 2B, Figure S5C and S5D, and Figure S6). qRT-PCR of the two different alternative splicing variants of DALRD3 showed that both variants are preferentially expressed in ERα positive cells and both reduced during E2 stimulation (Figure S3B and Figure S5C). These results highlight that E2 stimulation of the miR-191/425/DALRD3 transcriptional unit is essentially related to the production of miR-191 and miR-425. The reduction of the host gene isoform 1 may be explained with the mechanism proposed by Gromak *et al.* which showed that the cleavage of an intron can affect alternative splicing if it occurs between an alternatively spliced exon and its intronic regulatory elements [58]. Moreover, it has been demonstrated that ERα directly interacts with Drosha to modulate the processing of E2-regulated microRNAs [59]. In this scenario, we can hypothesize that the recruitment of ERα at the upstream promoter (Figure 2C) might improve the assembly of the Microprocessor complex at miR-191/425 locus and increase the cleavage of the intron for the production of the miRs, impairing the processing of the pre-mRNA. We further show that the increase of miR-191 and miR-425 upon E2 stimulation is associated with gradual reduction of polII accumulation on the downstream promoter (Figure 2C). Interestingly, this negative effect on DALRD3 promoter 2 is independent by ERα (silencing of ERα does not modify the downstream promoter activity), but is still related to E2 treatment, based on the strong reduction of promoter activity after E2 treatment (Figure 2E and Figure S6C). Both genomic and non-genomic estrogen actions may contribute to the regulation of miR-191/425-DALRD3 transcriptional unit [60], [61]: E2 treatment induces recruitment of ERα at the upstream promoter to improve only the accumulation of miR-191/425 (i.e., genomic regulation/processing activity), while estrogen-mediated effects, transmitted via enzymatic pathways or ion channels, induces repression of the downstream promoter (non-genomic regulation).

Next, we focused on the functional role of miR-191 and miR-425 in ERα signaling. Inhibition of miR-191 and miR-425 strikingly impairs cell proliferation and tumor formation in ERα positive cells (Figure 3A, 3D). Moreover, miR-19/425 overexpression in hormone deprived ERα positive cells, which have low levels of endogenous miR-191/425, reduces cell cycle arrest and apoptosis (Figure 3C). In silico analyses, based on the endonucleolytical activity of microRNAs, identify Early Growth Response 1 (EGR1) as a miR-191 target (Figure 3E, 3F and Figure S9A). EGR1 is involved in the regulation of cell growth and differentiation in response to signals, such as mitogens, growth factors, and stress stimuli [62], [63]. In most human tumors, such as breast cancer, fibrosarcoma, and glioblastoma, EGR1 is described to be a tumor suppressor gene [64]–[66]. In fact, re-expression of EGR1 in human tumor cells inhibits neoplastic transformation [63]. EGR1 represents also an important upstream gatekeeper of the p53 tumor suppressor pathway and many p53 downstream target genes, such as CDKN1A (p21), are dependent on EGR1 status. We demonstrate that during E2 stimulation, after an initial increase, the levels of EGR1 are repressed (Figure 3G and Figure S9B). Inhibition of miR-191 blocks the suppression of EGR1 and induces high levels of CDKN1A (p21) (Figure 3G, 3H) explaining at least in part the anti-proliferative activity of miR-191/425 cluster knockdown. However, the tumor-suppressive role of EGR1 seems to be tissue specific, because several studies implicated a tumor growth-promoting role of EGR1 in prostate cancer progression [67]–[69].

The loss of ERα expression causes tumor growth that is no longer under estrogen control, which leads to greater cancer aggressiveness and the failure of endocrine therapy. Therefore, restoration of ERα protein expression or signaling in ERα negative breast cancer cells represents an important key event to promote apoptosis and differentiation of aggressive breast cancer. Since miR-191 and miR-425 are players of the ERα signaling, we also inquire their role in ERα negative breast cancer. To this aim, we overexpressed both miRs in ERα negative cells and showed that miR-191 and miR-425 markedly alters the transcriptome of aggressive breast cancer cells, resulting in impaired tumor growth and metastasis (Figure 4 and Figure 5). Mechanistically, the effects of miR-191 and miR-425 on tumor growth and invasion require, at least in part, the suppression of SATB1, CCND2 and FSCN1. Specifically, miR-191-mediated SATB1 repression is associated with gain of epithelial markers (e.g., such as e-cadherin), and loss of mesenchymal markers (e.g., fibronectin and vimentin) (Figure 5C and Figure 6D). The increase of e-cadherin levels, mediated by miR-191/425, results in greater cell-cell adhesion, reduced detachment of cells, and cytoplasmic localization of β-catenin (Figure 5E). Mounting evidence indicates multiple reciprocal interactions of e-cadherin and cytoplasmic β-catenin with EMT-inducing transcriptional repressors to destabilize an invasive mesenchymal phenotype of epithelial tumor cells. Moreover, SATB1 and CCND2 repression by miR-191 are related to the suppression of the PI3K/AKT pathway and the corresponding reduced cell proliferation and tumor growth. We have also identified FSCN1, which is responsible for the reduced invasiveness and partial reversion to an epithelial morphology, as a target of miR-425 (Figure 6C).

All together our experiments demonstrate a duality in the biological role of miR-191/425 cluster in breast cancer: estrogen dependent-high levels of miR-191/425 induce proliferation in ERalpha positive cells by suppressing a strong tumor-suppressor gene, such as EGR1; low levels of miR-191/425 cluster are essential for the high expression of important modulators, such as SATB1, CCND2 and FSCN1, which confer a proliferative advantage to aggressive breast cancer cells.

## Materials and Methods

### Cell culture and tissue samples

Human breast cancer cell lines MCF10A, MCF10F, MCF7, T47D, BT474, BT483, ZR-75-1, MDA-MB361, HBL-100, SKBr3, MDA-MB-468, MDA-MB-453, BT549, MDA-MB-436, MDA-MB-231 as well as the Human Embryonic Kidney cell line HEK293, were purchased from the American Type Culture Collection (ATCC) and grown in accordance with ATCC recommendations. ERα, progesterone receptor (PGR) and HER2 status were confirmed for all cell lines by Western blot analyses. All transfections were carried out with Lipofectamine 2000 (Invitrogen, Carlsbad, CA) according to the manufacturer's instructions. For hormone depletion experiments, MCF7 cells were grown to 70% confluency in phenol red–free DMEM supplemented with 5% charcoal–dextran-stripped FBS for 6 days and collected every two days with the relative normal growth control. For estradiol (E2) treatments (Sigma Aldrich), MCF7 cells were hormone starved for 6 days and then treated with E2 (10 nM) at the indicated times. For Fulvestrant treatments, MCF7 cells were treated daily with fulvestrant (Sigma Aldrich) (100 nM) and collected at the reported time points. The 44 breast tumor tissue samples were provided from the Department of Pathology, The Ohio State University. All human tissues were obtained according to a protocol approved by the Ohio State Institutional Review Board.

### Taqman qRT–PCR and human microRNA card array

Quantitative real-time PCR (qRT-PCR) was performed with the TaqMan PCR Kit (Applied Biosystems, Foster City, CA), followed by the detection with the Applied Biosystems 7900HT Sequence Detection System (P/N: 4329002, Applied Biosystems). PCR was carried out in 10 µL of reaction buffer containing 0.67 µL RT product, 1 µL TaqMan Universal PCR Master Mix (P/N: 4324018, Applied Biosystems), 0.2 mM TaqMan probe, 1.5 mM forward primer, and 0.7 mM reverse primer. The reaction mixture was incubated in a 96-well plate at 95°C for 10 minutes, followed by 40 cycles of denaturation (95°C for 15 seconds) and extension (60°C for 1 minute). All reactions were performed in triplicate. Simultaneous quantification of small endogenous nucleolar RNA U44/U48 was used as a reference for TaqMan assay data normalization. For quantification of DALRD3, trefoil factor 1 (TFF1/pS2), pri-miR-191, pri-miR-425, VEGFA, FSCN1, EGR1, TNC, CDC42, SATB1, SOX4, CCND1, VIM, CCND2, E2F1, SIAH2, API5, FIBR, CSDA, MYC, CTNN1 and CDH1 mRNAs, the appropriate TaqMan probes were purchased from Applied Biosystems. The TaqMan Array Human MicroRNA Card (Applied Biosystem) Set v3.0 is a two-card set containing a total of 384 TaqMan MicroRNA Assays per card that enables accurate quantification of 754 human miRNAs. Included on each array are three TaqMan MicroRNA Assays as endogenous controls to aid in data normalization and one TaqMan MicroRNA Assay not related to human as a negative control.

### Microarray analyses

The hybridized Human Genome U133A 2.0 Array (Affymetrix) was scanned and analyzed with the Affymetrix Microarray Analysis Suite version 5.0. The average density of hybridization signals from three independent samples was used for data analysis, and genes with signal density less than 300 pixels were omitted from the analysis. P values were calculated with two-sided t-tests with unequal variance assumptions. To correct for multiple hypothesis testing, the false discovery rate was calculated. Differentially expressed genes were selected using both a false discovery rate of less than 0.01 and a fold-change greater than 1.5 or less than −1.5. A tree cluster was generated by hierarchical cluster analysis to classify the miR-transfected cells; for this analysis, we used average linkage metrics and centered Pearson correlation (Cluster 3.0). Java Treeview 1.1 (http://sourceforge.net/projects/jtreeview/) was used for tree visualization. The associations between gene modulations by two miRNAs were examined using a two-sided Fisher exact test. The association between modulations by any two miRNAs was statistically significant if P was less than .001. The online program Pathway-Express (http://vortex.cs.wayne.edu/Projects.html) was used to explore the most biologically relevant pathways affected by a list of input genes. Specific biological pathways were defined by the Kyoto Encyclopedia of Genes and Genomes database (Kanehisa Laboratories, Kyoto, Japan) (http://www.genome.jp/kegg/pathway.html). Pathways were considered statistically significant if the corrected gamma P was less than 0.01.

### Cell cycle analyses

For cell-cycle analysis, MDA-MB-231 and MDA-MB-436 cells were plated in 6 cm dishes, transfected as indicated in the figures, trypsinized, washed in PBS, and fixed with ice-cold 70% ethanol while vortexing. Cells were rehydrated in PBS and stained 30 min at RT with propidium iodide (50 mg/ml PI, 0.5 mg/ml RNase in PBS) prior to flow-cytometric analysis. Lenti-GFP, lenti-191 and lenti-425 infected-cells were also analyzed by flow cytometry after 12 h treatment with nocodazole.

### In vivo experiments

All mouse experiments were conducted following protocols approved by the institutional animal care and use committee at the Ohio State University. Parental MDA-MB-231, lenti-GFP, lenti-191 and lenti-425 infected-cells (5×10^6^) were injected subcutaneously into the right flank of 6-week-old athymic nude mice. Tumor size was assessed twice per week using a digital caliper. Tumor volumes were determined by measuring the length (l) and the width (w) of the tumor and calculating the volume (V = lw^2^/2). Statistical significance between the control and treated mice was evaluated using Student's t test. We sacrifiedthe mice 35 days after injection and tumors were excided and processed for histology and for RNA and protein extractions. 4 µm sections of tumor tissues were stained with hematoxylin/eosin and with Ki-67 by immunohistochemistry. For MCF7 and ZR-75-1 xenografts, estradiol pellets (Innovative Research) were implanted in nude mouse and after two weeks mice were injected subcutaneously with one 10 cm plate of anti-miR191/425 transfected MCF7 or ZR-75-1 cells. Mouse experiments were conducted after approval by the institutional animal care and use committee at Ohio State University.

### Migration assay

Transwell insert chambers with an 8-µm porous membrane (Greiner Bio One) were used for the assay. Cells were washed three times with PBS and added to the top chamber in serum-free medium. The bottom chamber was filled with medium containing 10% FBS. Cells were incubated for 24 h at 37°C in a 5% CO2 humidified incubator. To quantify migrating cells, cells in the top chamber were removed by using a cotton-tipped swab, and the migrated cells were fixed in PBS, 25% glutaraldehyde and stained with crystal violet stain, visualized under a phase-contrast microscope and photographed. Crystal-violet–stained cells were then solubilized in acetic acid and methanol (1∶1), and absorbance was measured at 595 nm. For the scratch assay, parental MDA-MB-231 cells, lenti-GFP, lenti-miR191 and lenti-miR425 infected-cells were plated in culture dishes and after 24 h the confluent monolayer was scratched. Images were acquired directly after scratching (0 h) and after 5 h, 9 h and 16 h. For quantification of migration distance Image J software was used. The distance covered was calculated by converting pixel to millimeters.

### miRNA locked nucleic acid in situ hybridization

In situ hybridization (ISH) was carried out on deparaffinized human breast tissues using previously published protocol (Nuovo GJ, 2009), which includes a digestion in pepsin (1.3 mg/ml) for 30 minutes. The probes contained the dispersed locked nucleic acid (LNA) modified bases with digoxigenin conjugated to the 5′ end. The probe cocktail and tissue miRNA were co-denatured at 60°C for 5 minutes, followed by hybridization at 37°C overnight and a stringency wash in 0.2× SSC and 2% bovine serum albumin at 4°C for 10 minutes. The probe-target complex was seen due to the action of alkaline phosphatase on the chromogen nitroblue tetrazolium and bromochloroindolyl phosphate (NBT/BCIP). Negative controls included the use of a probe that should yield a negative result in such tissues (scrambled miRNA).

### RNA extraction and Northern blotting

Total RNA isolation was performed with Trizol (Invitrogen, Carlsbad, CA) according to the manufacturer's instructions. For, acrylamide northern blotting 10 µg aliquots of total RNA were resolved on a 15% denaturing polyacrylamide gel (Bio-Rad, Hercules, CA) and were electrophoretically transferred to BrightStar blotting membrane (Ambion Inc, Austin, TX). The oligonucleotide encoding the complementary sequence of the mature miRNA annotated in the miRNA Registry (release 14: September 2009) was end-labeled with [γ^32^ P]-ATP by T4 polynucleotide kinase (USB, Cleveland, OH). RNA-blotted membrane was prehybridized in Ultrahyb Oligo solution (Ambion Inc) and subsequently hybridized in the same solution containing probe at a concentration of 10^6^ cpm/mL at 37°C overnight. The membrane was washed at high stringency in the solution containing 2× standard saline citrate and 1% sodium dodecyl sulfate at 37°C. Northern hybridization signals were captured and converted to digital images with the Typhoon Scanner (GE Healthcare Biosciences, Piscataway, NJ).

### Chromatin immunoprecipitation assay

Chromatin immunoprecipitation (ChIP) assays were performed with the ChIP assay kit (Upstate Biotechnology, Lake Placid, NY) with minor modifications. Briefly, MCF7 and MDA-MB-436 cells were hormone starved for 6 days and then treated with E2 (10 nM) for 3 h, 6 h and 24 h. The cross-linking was performed with 1% formaldehyde at 37°C for 10 minutes. Cells were then rinsed with ice-cold PBS and resuspended in 0.4 mL of lysis buffer containing 1% sodium dodecyl sulfate, 10 mM EDTA, 50 mM Tris–HCl, pH 8.1, 1× protease inhibitor cocktail (Roche Molecular Biochemicals), and sonicated. A 30 µL aliquot of the preparation was treated to reverse the cross-linking, deproteinized with proteinase K, extracted with phenol–chloroform, and the DNA concentration determined by Nanodrop 2000c (Thermo Scientific, Wilmington, DE) measurements. An aliquot of chromatin preparation containing 25 µg DNA was used per ChIP. The primary antibodies used for immunoprecipitation were rabbit polyclonal ERα (Bethyl Laboratories [Montgomery, TX] A300-498A), rabbit IgG control (Zymed, Carlsbad, CA), rabbit polyclonal acetyl-H3 (Upstate Biotechnology), rabbit polyclonal polIII (Upstate Biotechnology). ChIP-enriched DNA was subjected to SYBR green qPCR (Applied Biosystems). Primer sequences are listed in the Primer Table. Results were expressed as relative enrichment according to the following formula: 2^−[(ctChIP−ctinput)−(ctIgG−ctinput)]^, where ct_ChIP_, ct_IgG_, and ct_input_ indicate the cycle threshold for the specific antibody, IgG control, and input (5% of the total amount of immunoprecipitated material), respectively.

### Transcriptional elements analyses

For miR-191 and -425 promoter prediction, a 9200 base pair (bp) DNA genomic region spanning miR-191 and-425 was used as input for the online software Promoter 2.0 (http://www.cbs.dtu.dk/services/promoter/).

### Plasmid construction

To generate SATB1, CCN2, CTDSP2, SOX4, LRCC8A, SLC16A2, EGR1, CSDA, FSCN1, TNC, SIAH2 and CSDA luciferase reporter constructs, the 3′UTRs were amplified by polymerase chain reaction (PCR) and cloned downstream of the luciferase-coding sequence in the pGL3-control vector at the XbaI restriction site (Promega). Mutations were introduced into the miRNA-binding sites by using the QuikChange Mutagenesis Kit (Stratagene, La Jolla, CA). To map the miR-191-425 promoter, prom1 or prom2 genomic region (see schematic representation of miR-191/425-DARLD3 transcription unit, Figure 3, C) were amplified by PCR and cloned at the NheI and XhoI sites of the pGL3-basic vector (Promega). All constructs were sequenced to verify integrity.

### Luciferase assay for target and promoter identification

To confirm that SATB1, CCN2, CTDSP2, SOX4, LRCC8A, SLC16A2, EGR1, CSDA, FSCN1, TNC, SIAH2, CSDA harbor responsive seed regions (complementary sequences) so that miR-191 and/or miR-425 can bind to their 3′UTRs, 250 ng of pGL3 reporter vector carrying the miR-191 or miR-425 binding site (see plasmid construct, Figure S9A), 25 ng of the phRL-SV40 control vector (Promega), and 100 nM miRNA precursors or scrambled sequence miRNA control (Ambion, Inc, Austin, TX) were cotransfected into HEK293 cells in 24-well plates. To map the miR-191 and miR-425 promoter, 250 ng of pGL3 reporter vector carrying prom1 or prom2 genomic region (see schematic representation of miR-191/425-DARLD3 transcription unit, Figure 3, C) and 25 ng of the phRL-SV40 control vector were cotransfected into HEK293 cells in 24-well plates. To asses estrogen responsiveness of the two promoter regions, same experiment was carried out in 5 breast cancer cell lines with different ERalpha status, in MCF7 cells after E2 (10 nM) treatment and in MCF7 after ERalpha silencing. Firefly luciferase activity was measured with a Dual Luciferase Assay Kit (Promega) 24 hours after transfection and normalized with a Renilla luciferase reference plasmid. Reporter assays were carried out in quadruplicate. Statistical significance was analyzed by the unpaired Student t test.

### Western blotting

All cell lysates were prepared by using RadioImmuno Precipitation Assay Buffer (Pierce, Rockford, IL). Fifty micrograms of cell lysates was separated by sodium dodecyl sulfate–polyacrylamide gel electrophoresis and then electroblotted onto a polyvinylidene fluoride membrane (Hybond P; Amersham Biosciences, Piscataway, NJ). All primary antibodies used for western blot analyses are reported in Supplemental Materials and Methods (available online). Detection was performed with horseradish peroxidase–conjugated secondary antibodies (specific to rabbit and mouse) and enhanced chemiluminescence (Pierce).

### Nuclear/cytoplasmic differential protein extraction

Nuclear/Cytoplasmic differential protein extraction was performed by using the NE-PER Nuclear and Cytoplasmic extraction kit (Pierce) according to the manufacturer's instructions.

### Generation of stable clones with miR-191 and miR-425 overexpression

MDA-MB-231 cells were stably infected with the Human pre-microRNA Expression Construct Lenti-miR expression plasmid containing the full-length miR-191 or miR-425 and the GFP gene under the control of two different promoters (System Biosciences). An empty vector was used as control. Pre-miRs expression and control constructs were packaged with pPACKH1 Lentivector Packaging Plasmid mix (System Biosciences) in a 293TN packaging cell line. Viruses were concentrated using PEGit Virus Precipitation Solution, and titers were analyzed using the UltraRapid Lentiviral Titer Kit (System Biosciences). Infected cells were selected by FACS analysis (FACScalibur; BD Bioscience). Infection efficiency >90% was verified by fluorescent microscopy and confirmed by real-time PCR for miRs expression.

### Proliferation assays

MDA-MB-231 cells, previously transfected with miR-191 or miR-425 precursors for 72 h, were plated (3000 per well) in 96-well plates and grown for 96 hours after transfection (final miRNA concentration of 100 nM) in normal culture conditions. MCF7 in normal culture conditions (+E2) transfected with anti-miR-191/425 and CTR oligonucleotide or in hormon deprivation conditions (−E2) transfected with miR-191/425 and CTR oligonucleotide were plated in 96-well plates and grown for 96 hours after transfection. Cell proliferation was documented every 24 hours for 4 days using a 3-(4,5-dimethylthiazol-2-yl)-2,5-diphenyltetrazolium bromide assay kit (Promega, Madison, WI), and absorbance at 490 nm was evaluated by a SpectraMax 190 microplate reader (Molecular Devices, Sunnyvale, CA).

## Supporting Information

Figure S1 miR-191/425 genomic locus. Schematic representation of the human (A) and murine (B) genomic locus of miR-191/425 cluster. miRNAs are represented with red lines. Green boxes represent the CpG islands. Arrowheads indicate the direction of the transcription.
(TIF)

Figure S2 Co-expression of miR-191 and miR-425 with their host gene, DALRD3, in normal tissues and breast cancer cells. (A) Quantitative RT-PCR on mature miR-191, miR-425 and DALRD3 mRNA levels in 20 normal human tissues. (B) XY scatter plots to define the correlation between miR-191/425/DALRD3 expression in human normal tissues. (C) Expression levels of miR-191 and miR-425 in human breast cancer cells by qRT-PCR. All error bars indicate s.d.
(TIF)

Figure S3 Expression of DALRD3 mRNA in breast cancer specimens and cancer cells. (A) DALRD3 transcript expression with different probes in breast tumor subtypes from Oncomine analysis. The first author and statistical significance are indicated. (B) SYBR qRT-PCR to discriminate the expression levels of the two main splicing variants of DALRD3 in 15 breast cancer cells. Isoform1 represents the splicing variants that may be responsible for the transcription of miR-191/425 cluster.
(TIF)

Figure S4 miR-191 and miR-425 in situ hybridization (ISH) in human breast cancer. (A) In situ hybridization analysis of miR-191 and miR-425 expression in breast cancer tissues with different ERα expression status. Bars represent 200 µm. Two different cores for each microRNA and scrambled control oligonucleotide are represented for each category. Results are reported in the table as a percentage of the total number of ERα positive and ERα negative cores. (B) Co-labeling for miR-191 and miR-425 in human ERα positive breast tissue. Large and small arrows indicate tumor and stroma cells, respectively.
(TIF)

Figure S5 miR-191/425 and estrogen regulation. (A) qRT-PCR on TFF1/pS2 and mature miR-17 upon E2 (10 nM) stimulation. MCF7 cells were hormone starved for 6 days and treated daily with estrogen for 72 h. (B) qRT-PCR on the primary precursor of mir-191 and miR-425 after E2 (10 nM) stimulation. (C) qRT-PCR for both splicing variant1 ad 2 of DALRD3 after hormone stimulation of MCF7 cells. (D) qRT-PCR for total DALRD3, splicing variants1 and 2, and TFF1/pS2 after hormone starvation of MCF7 cells (NT: untreated; HS: hormone starved). Error bars indicate s.d. and * represent p-value<0.05 obtained with two-sided Student's t-test.
(TIF)

Figure S6 Fulvestrant treatment reduces miR191/425 levels. ERα positive cells, MCF7, were treated daily with fulvestrant (100 nM) and collected at the reported time point. (A) Western blot analyses to control ERα degradation after 72 h of fulvestrant treatment. GAPDH levels were used as a loading control. (B) miR-191/425 levels were assessed after 72 h of fulvestrant treatment by qRT-PCR. (C) qRT-PCR was used to define the levels of DALRD3 and TFF1/pS2 expression during fulvestrant treatment. Error bars indicate s.d. and * represents p-value<0.001 obtained with two-sided Student's t-test.
(TIF)

Figure S7 miR-191/425-DALRD3 promoter identification. (A) In silico analyses (http://www.cbs.dtu.dk/services/Promoter/) for the identification of the promoter elements related to miR-191/425-DALRD3 genomic DNA sequence. Outputs are reported in the table and represent the prediction for a transcription start site occurring within 100 base pairs upstream from that position. (B) Luciferase assay for prom1 and prom2 luciferase plasmids in 5 breast cancer cells with different ERα status. (C) Luciferase assay for prom1 and prom2 luciferase plasmids in ERα positive MCF7 cells after silencing of ERα. MCF7 were transfected with siRNA against ERα and scrambled siRNA control (100 nM). 48 h after transfection cells were transfected again with prom1 and prom2 plasmids and luciferase experiments were carried out 24 h after. Results for the luciferase assay are presented as an average of three independent experiments: error bars indicate s.d. and * represents p-value<0.001 obtained with two-sided Student's t-test.
(TIF)

Figure S8 miR-191/425 proliferative effect in ERα positive breast cancer cells. (A) Cell cycle analyses of ZR-75-1 cells transfected with anti miR-191/425 and scrambled control (CTR) oligonucleotide in normal culture condition. Cells were harvested 72 h following transfection, fixed, stained with propidium iodide, and analyzed by flow cytometry; the data are representative of three independent experiments. (B) In vivo growth kinetic of ZR-75-1 cells transfected with anti-miR-191/425 and scrambled control oligonucleotide. Briefly, ZR-75-1 were transfected in 10 cm plates by using 2-O-methyl anti miR-191 and miR-425 oligonucleotides (100 nM); 48 h after transfection, cells were detached and injected in nude mice previously implanted (two weeks before injection) with estradiol pellets. Images show average-sized tumors for each group. p-value was calculated on one experiment performed with 5 mice for each group.
(TIF)

Figure S9 E2 modulated targets of miR-191 and miR-425. (A) Intersection of predicted miR-191, miR-425 human targets and E2 repressed genes in MCF7 and ZR-75-1 cells. Only commonly modulated target genes are reported in the gray boxes. (B) qRT-PCR for EGR1 mRNA after E2 stimulation in MCF7 cells. Gene expression levels are reported as relative expression to GAPDH levels. Error bars indicate s.d. * represent p-value of 0.05. (C) qRT-PCR for miR-191 after E2 stimulation in anti-miR191 and scrambled control oligonucleotide transfected MCF7 cells. Gene expression levels are reported as relative expression to GAPDH levels. Error bars indicate s.d.
(TIF)

Figure S10 miR-191 and miR-425 signature in aggressive breast cancer cells. miR-191, miR-425 and scrambled control were transfected in MDA-MB-231 and cells were collected 72 h after transfection for genome-wide expression analyses. Differentially expressed genes (fold change >1.2 and p-value<0.001) are represented in the hierarchical tree and the modulated biological pathways are enlisted based on the Impact factor strength of miR-activated (blue) and repressed (yellow) genes.
(TIF)

Figure S11 Representation of miR191/425 binding sites of the target genes. (A) Schematic representation of miR191 and miR425 binding sites located in the 3′UTR (in green) of the target genes. The blue line defines the 3′UTR fragment cloned into pGL3 control plasmid and the arrowhead indicates the position of the miRNA binding site, whose sequence is reported below with the mutagenesis strategy used to generate the disruption between miRNA and mRNA of the target gene. (B) Luciferase assays for wildtype and disrupted miRNA binding sites of all target genes with increased luciferase activity after miR191 or miR425 enforced expression on mutated plasmid. Luciferase activity values for wildtype and mutated plasmids are represented as a ratio between the relative luciferase activity of miR-transfected cells with the relative luciferase activity of control-transfected cells.
(TIF)

Figure S12 miR-191/425 impair tumorigenicity of aggressive breast cancer cells. (A) MTT assay revealed a reduced growth rate in miR-191 and miR-425 overexpressing MDA-MB-436 cells compared to scrambled control cells. Error bars indicate s.d. and asterisks indicate p-value<0.05. (B) qRT-PCR to verify miR-191 and miR-425 overexpression in lenti-infected MDA-MB-231 or MDA-MB-436. Error bars indicate s.d. (C,D) 2D colonies formation assay in MDA-MB-436 stable cell line expressing miR-191 or miR-425 from lentiviral expression vectors, compared to the corresponding GFP control cells. Colony counting was performed by using the GS-800™ calibrated densitometer. Error bars indicate s.d. and asterisks indicate p-value<0.05. (E) Soft agar assay in which cells were seeded at a density of 4×10^3^ cells per 35-mm dish and cultured in 0.35% soft agar in RPMI 10% FBS at 37°C for 21 days. Colonies were stained with 0.05% crystal violet. Colony numbers in the entire dish were counted by using the GS-800 calibrated densitometer. Error bars indicate s.d. and asterisks indicate p-value<0.05. (F) Cell cycle analyses of MDA-MB-436 transiently-transfected cells. Cells were harvested 72 h following transfection, fixed, stained with propidium iodide and analyzed by flow cytometry. The data obtained were analyzed using ModFit software. Cells in G1 and in G2 phase of cell cycle are reported in red, cells in S phase are indicated with white and blue bars. Flow cytometry plots are representative of three independent experiments. (G) Lenti-viral infected MDA-MB-231 were analyzed by propidium iodide staining after 100 ng/mL nocodazole treatments for 16 h, before cells were released and harvested for FACS analysis. The data obtained were analyzed using ModFit software. Cells in G1 and in G2 phase of cell cycle are reported in red, cells in S phase are indicated with white and blue bars. Flow cytometry plots are representative of three independent experiments. (H) miR-191/425 modulated targets were analyzed by Western blot analyses 72 h after miR-191 and miR-425 overexpression in MDA-MB-436. Representative Western blots are shown.
(TIF)

Figure S13 miR-191/425 in vivo effects. (A) Hematoxylin-eosin of subcutaneous MDA-MB-231 lentiGFP-infected tumors. Arrows in panels indicate areas of tumor invasion in the muscle cells of the fibrotic capsule. (B) qRT-PCR to verify the expression of miR-191 and miR-425 in the resected xenografted tumors. (C) Expression levels of fibronectin, e-cadherin and beta-catenin were determined by taqman qRT-PCR in xenografted tumors. Error bars indicate s.d. (* indicates p-value<0.01; ** indicates p-value = 0.011). (D) Densitometric analyses of the Western blots presented in Figure 5H.
(TIF)

Figure S14 miR-191/425 impair motility of MDA-MB-436 breast cancer cells. (A) Transwell motility assay was performed by plating miR191,-425 and scrambled control transfected MDA-MB-436 cells on inserts. Collected data from three separate experiments performed in triplicate are represented as number of cells per field. (B) Wound healing assay done on the miR-191,-425 stable clones and GPF control cells MDA-MB-436. The diameters of wounds were measured on the microscopic photos at 0, 5 h, 9 h and 16 h after wounding. Changes in wound diameter were computed into percentage to represent wound closure. Error bars indicate s.d.
(TIF)

Table S1 microRNA differentially expressed after hormone starvation and estradiol stimulation. Taqman multiplex miRNA cards results of hormone starvation and E2 stimulation of MCF7 cells. All microRNAs represented have a p-value<0.05 and a fold change >1.2.
(XLS)

Table S2 Gene expression signature of miR-191 and miR-425 in MDA-MB-231 cells. All modulates genes with a fold change and p-value higher of 1.5 and 0.05 were reported. Modulated targets were obtained by comparison between miR-down-modulated gene list and Tagetscan predicted targets.
(XLS)
