# Correction: Estrogen Mediated-Activation of miR-191/425 Cluster Modulates Tumorigenicity of Breast Cancer Cells Depending on Estrogen Receptor Status

**DOI:** 10.1371/annotation/92dfa670-d431-4d68-b70b-706df1f93e46

**Published:** 2013-04-25

**Authors:** Gianpiero Di Leva, Claudia Piovan, Pierluigi Gasparini, Apollinaire Ngankeu, Cristian Taccioli, Daniel Briskin, Douglas G. Cheung, Brad Bolon, Laura Anderlucci, Hansjuerg Alder, Gerard Nuovo, Meng Li, Marilena V. Iorio, Marco Galasso, Santhanam Ramasamy, Guido Marcucci, Danilo Perrotti, Kimerly A. Powell, Anna Bratasz, Michela Garofalo, Kenneth P. Nephew, Carlo M. Croce

The fifteenth author's name was listed incorrectly. The correct name is: Ramasamy Santhanam

