# Supplementary figures and images for: Estrogen Mediated-Activation of miR-191/425 Cluster Modulates Tumorigenicity of Breast Cancer Cells Depending on Estrogen Receptor Status
Source: PLoS Genet. 2013 Mar 7;9(3):e1003311. doi: 10.1371/journal.pgen.1003311 (PMC3591271; doi:10.1371/journal.pgen.1003311)

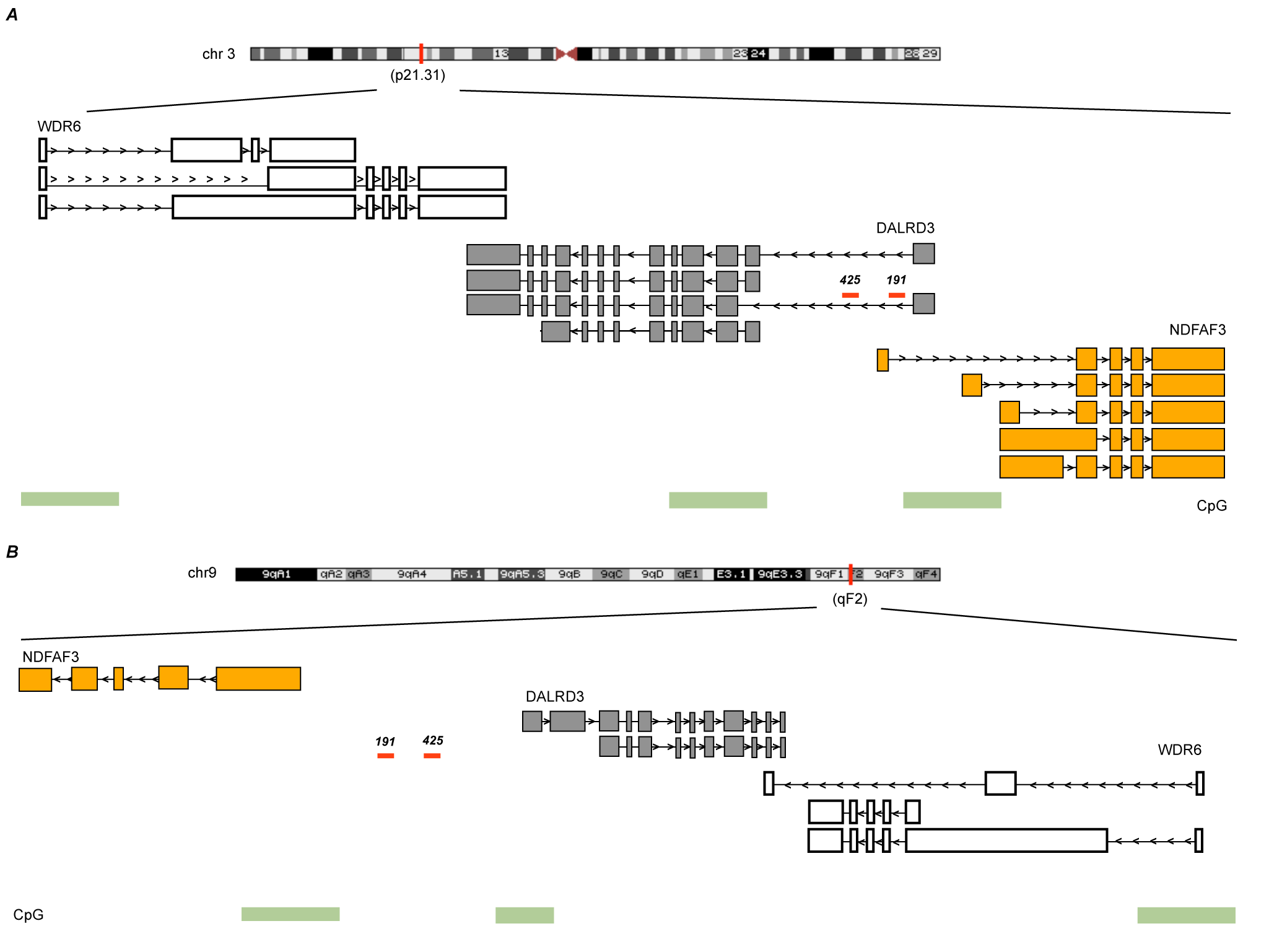

Supplement: Figure S1 — miR-191/425 genomic locus. Schematic representation of the human (A) and murine (B) genomic locus of miR-191/425 cluster. miRNAs are represented with red lines. Green boxes represent the CpG islands. Arrowheads indicate the direction of the transcription. (TIF) [file pgen.1003311.s001.tif]

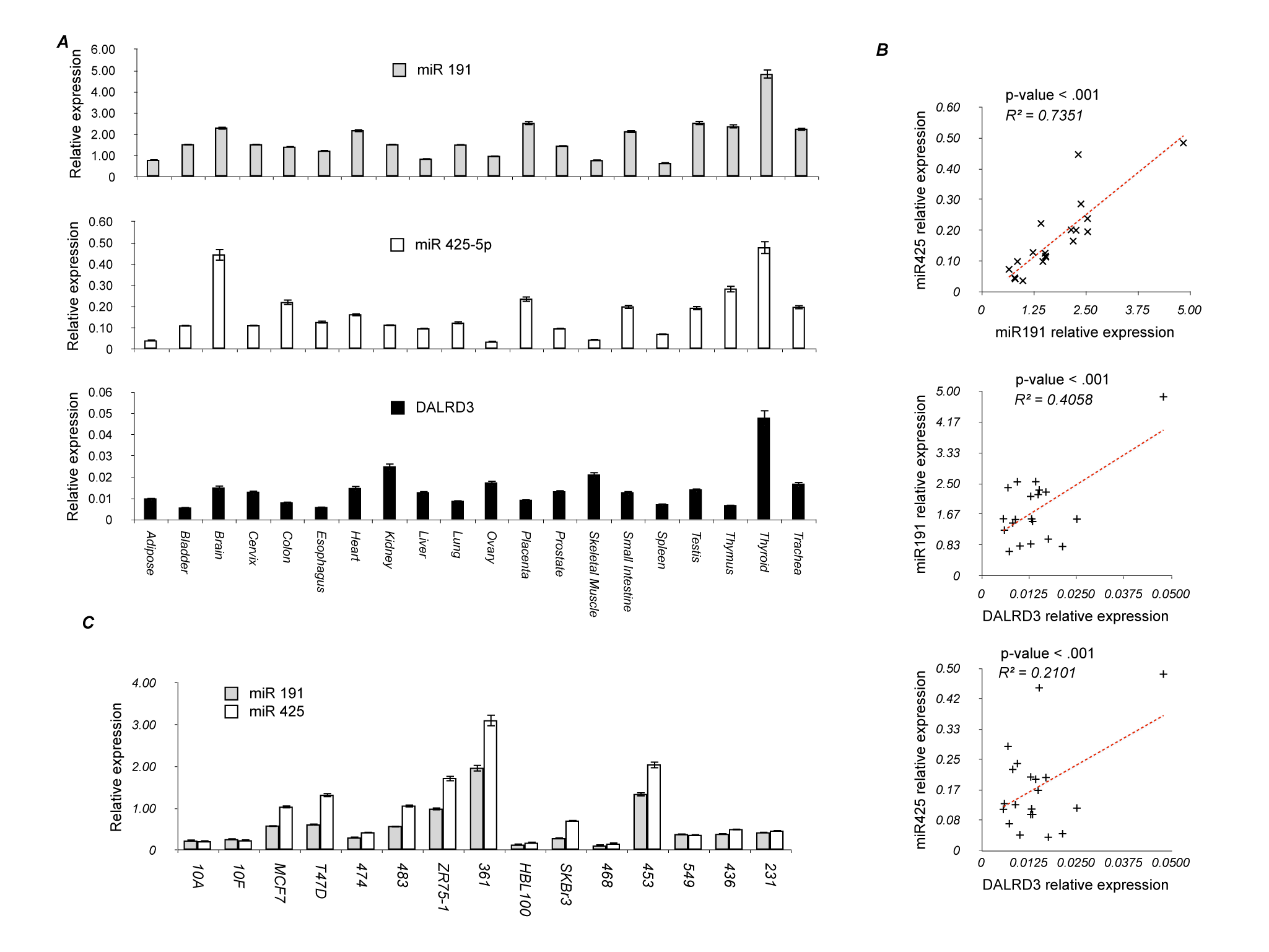

Supplement: Figure S2 — Co-expression of miR-191 and miR-425 with their host gene, DALRD3, in normal tissues and breast cancer cells. (A) Quantitative RT-PCR on mature miR-191, miR-425 and DALRD3 mRNA levels in 20 normal human tissues. (B) XY scatter plots to define the correlation between miR-191/425/DALRD3 expression in human normal tissues. (C) Expression levels of miR-191 and miR-425 in human breast cancer cells by qRT-PCR. All error bars indicate s.d. (TIF) [file pgen.1003311.s002.tif]

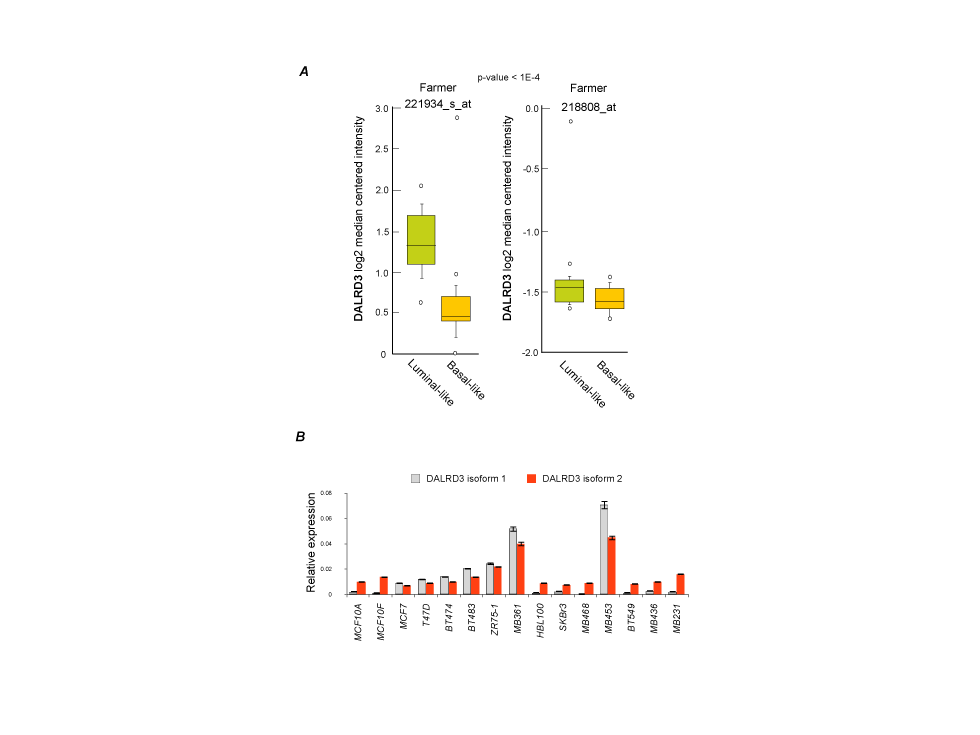

Supplement: Figure S3 — Expression of DALRD3 mRNA in breast cancer specimens and cancer cells. (A) DALRD3 transcript expression with different probes in breast tumor subtypes from Oncomine analysis. The first author and statistical significance are indicated. (B) SYBR qRT-PCR to discriminate the expression levels of the two main splicing variants of DALRD3 in 15 breast cancer cells. Isoform1 represents the splicing variants that may be responsible for the transcription of miR-191/425 cluster. (TIF) [file pgen.1003311.s003.tif]

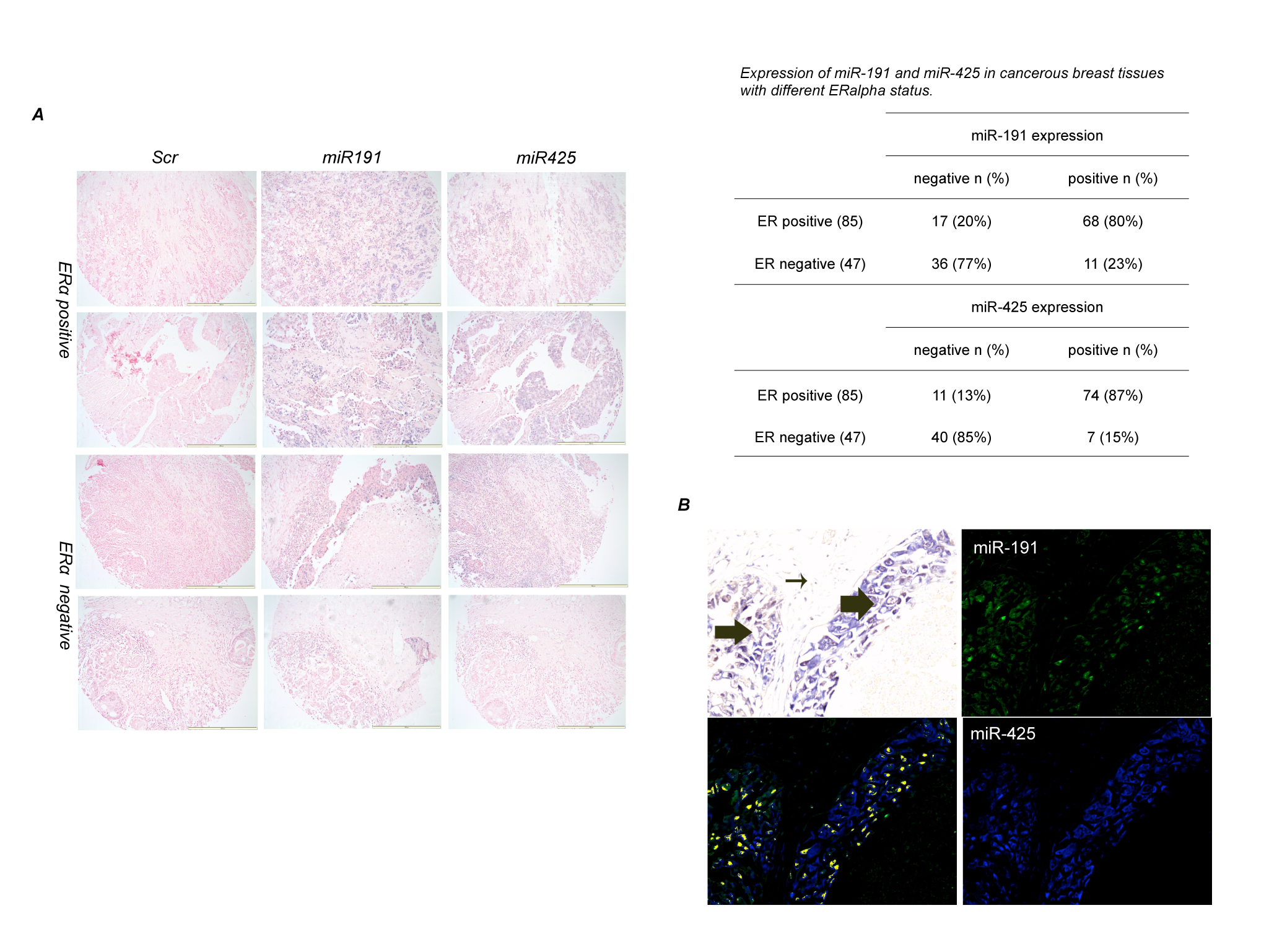

Supplement: Figure S4 — miR-191 and miR-425 in situ hybridization (ISH) in human breast cancer. (A) In situ hybridization analysis of miR-191 and miR-425 expression in breast cancer tissues with different ERα expression status. Bars represent 200 µm. Two different cores for each microRNA and scrambled control oligonucleotide are represented for each category. Results are reported in the table as a percentage of the total number of ERα positive and ERα negative cores. (B) Co-labeling for miR-191 and miR-425 in human ERα positive breast tissue. Large and small arrows indicate tumor and stroma cells, respectively. (TIF) [file pgen.1003311.s004.tif]

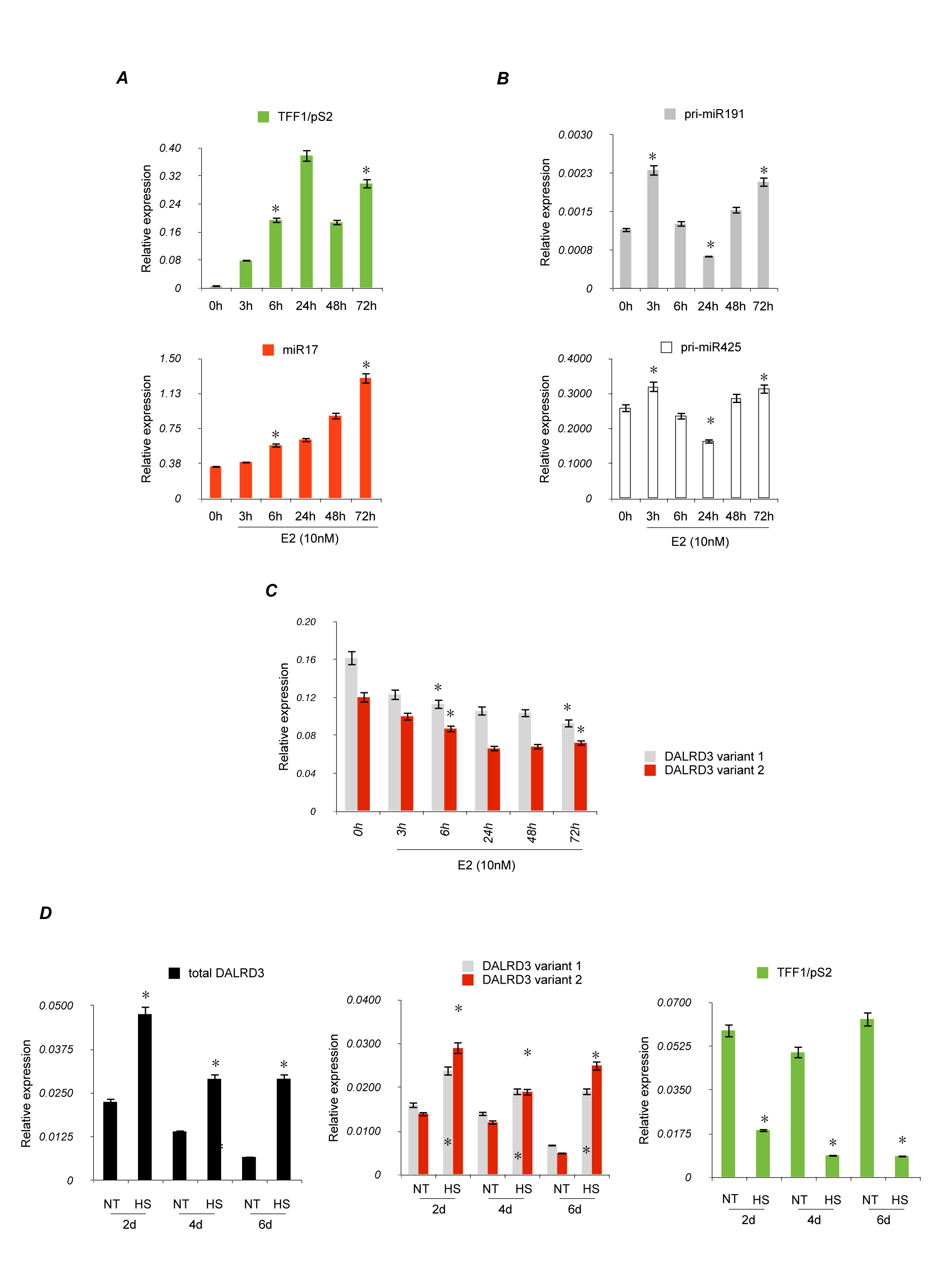

Supplement: Figure S5 — miR-191/425 and estrogen regulation. (A) qRT-PCR on TFF1/pS2 and mature miR-17 upon E2 (10 nM) stimulation. MCF7 cells were hormone starved for 6 days and treated daily with estrogen for 72 h. (B) qRT-PCR on the primary precursor of mir-191 and miR-425 after E2 (10 nM) stimulation. (C) qRT-PCR for both splicing variant1 ad 2 of DALRD3 after hormone stimulation of MCF7 cells. (D) qRT-PCR for total DALRD3, splicing variants1 and 2, and TFF1/pS2 after hormone starvation of MCF7 cells (NT: untreated; HS: hormone starved). Error bars indicate s.d. and * represent p-value<0.05 obtained with two-sided Student's t-test. (TIF) [file pgen.1003311.s005.tif]

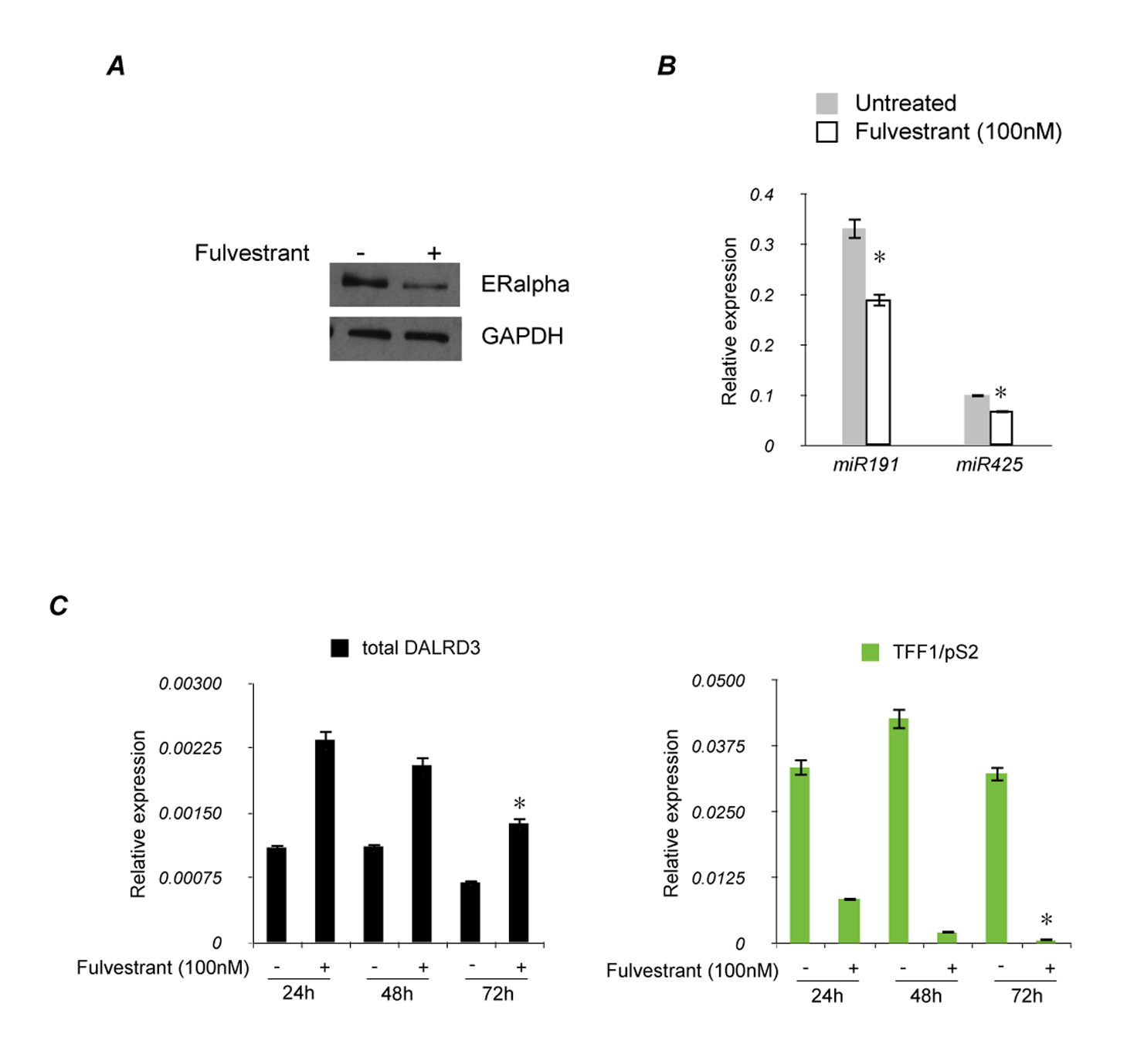

Supplement: Figure S6 — Fulvestrant treatment reduces miR191/425 levels. ERα positive cells, MCF7, were treated daily with fulvestrant (100 nM) and collected at the reported time point. (A) Western blot analyses to control ERα degradation after 72 h of fulvestrant treatment. GAPDH levels were used as a loading control. (B) miR-191/425 levels were assessed after 72 h of fulvestrant treatment by qRT-PCR. (C) qRT-PCR was used to define the levels of DALRD3 and TFF1/pS2 expression during fulvestrant treatment. Error bars indicate s.d. and * represents p-value<0.001 obtained with two-sided Student's t-test. (TIF) [file pgen.1003311.s006.tif]

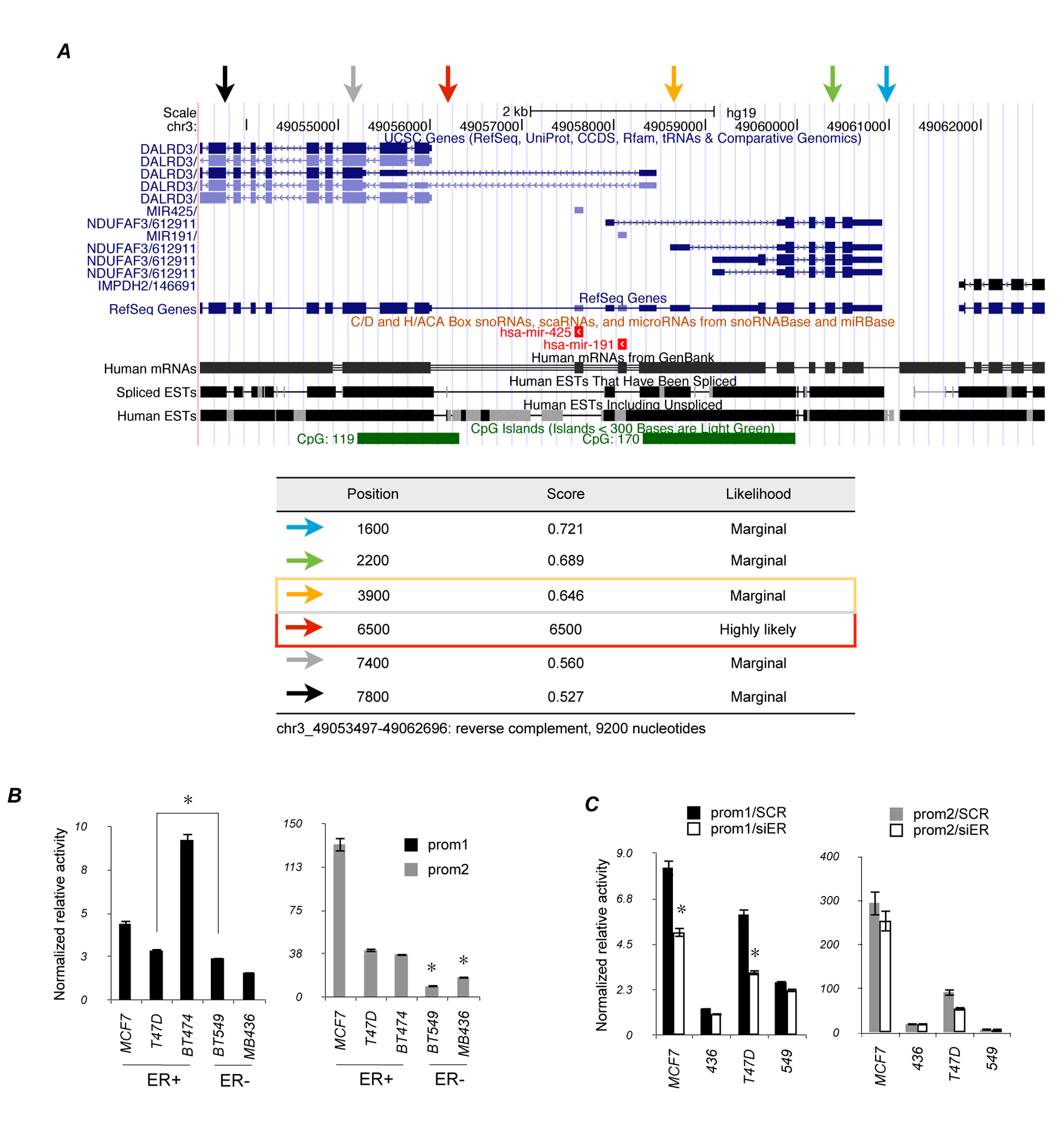

Supplement: Figure S7 — miR-191/425-DALRD3 promoter identification. (A) In silico analyses (http://www.cbs.dtu.dk/services/Promoter/) for the identification of the promoter elements related to miR-191/425-DALRD3 genomic DNA sequence. Outputs are reported in the table and represent the prediction for a transcription start site occurring within 100 base pairs upstream from that position. (B) Luciferase assay for prom1 and prom2 luciferase plasmids in 5 breast cancer cells with different ERα status. (C) Luciferase assay for prom1 and prom2 luciferase plasmids in ERα positive MCF7 cells after silencing of ERα. MCF7 were transfected with siRNA against ERα and scrambled siRNA control (100 nM). 48 h after transfection cells were transfected again with prom1 and prom2 plasmids and luciferase experiments were carried out 24 h after. Results for the luciferase assay are presented as an average of three independent experiments: error bars indicate s.d. and * represents p-value<0.001 obtained with two-sided Student's t-test. (TIF) [file pgen.1003311.s007.tif]

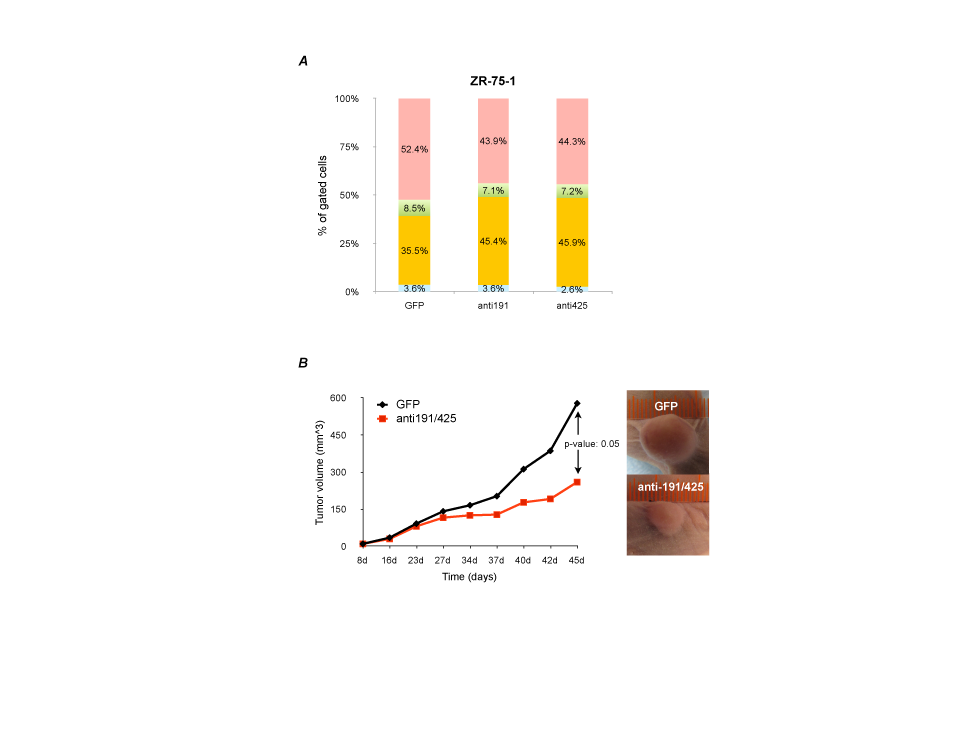

Supplement: Figure S8 — miR-191/425 proliferative effect in ERα positive breast cancer cells. (A) Cell cycle analyses of ZR-75-1 cells transfected with anti miR-191/425 and scrambled control (CTR) oligonucleotide in normal culture condition. Cells were harvested 72 h following transfection, fixed, stained with propidium iodide, and analyzed by flow cytometry; the data are representative of three independent experiments. (B) In vivo growth kinetic of ZR-75-1 cells transfected with anti-miR-191/425 and scrambled control oligonucleotide. Briefly, ZR-75-1 were transfected in 10 cm plates by using 2-O-methyl anti miR-191 and miR-425 oligonucleotides (100 nM); 48 h after transfection, cells were detached and injected in nude mice previously implanted (two weeks before injection) with estradiol pellets. Images show average-sized tumors for each group. p-value was calculated on one experiment performed with 5 mice for each group. (TIF) [file pgen.1003311.s008.tif]

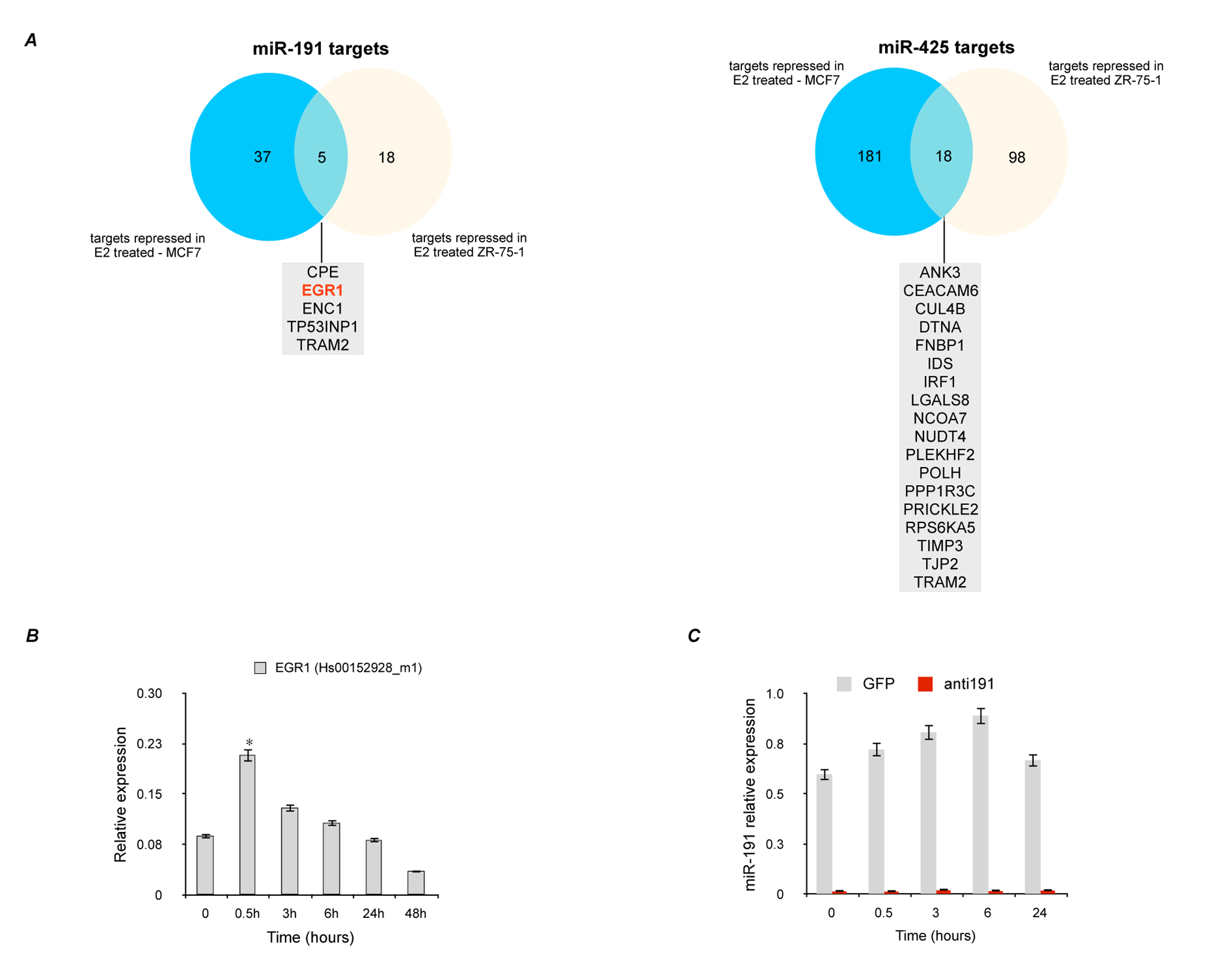

Supplement: Figure S9 — E2 modulated targets of miR-191 and miR-425. (A) Intersection of predicted miR-191, miR-425 human targets and E2 repressed genes in MCF7 and ZR-75-1 cells. Only commonly modulated target genes are reported in the gray boxes. (B) qRT-PCR for EGR1 mRNA after E2 stimulation in MCF7 cells. Gene expression levels are reported as relative expression to GAPDH levels. Error bars indicate s.d. * represent p-value of 0.05. (C) qRT-PCR for miR-191 after E2 stimulation in anti-miR191 and scrambled control oligonucleotide transfected MCF7 cells. Gene expression levels are reported as relative expression to GAPDH levels. Error bars indicate s.d. (TIF) [file pgen.1003311.s009.tif]

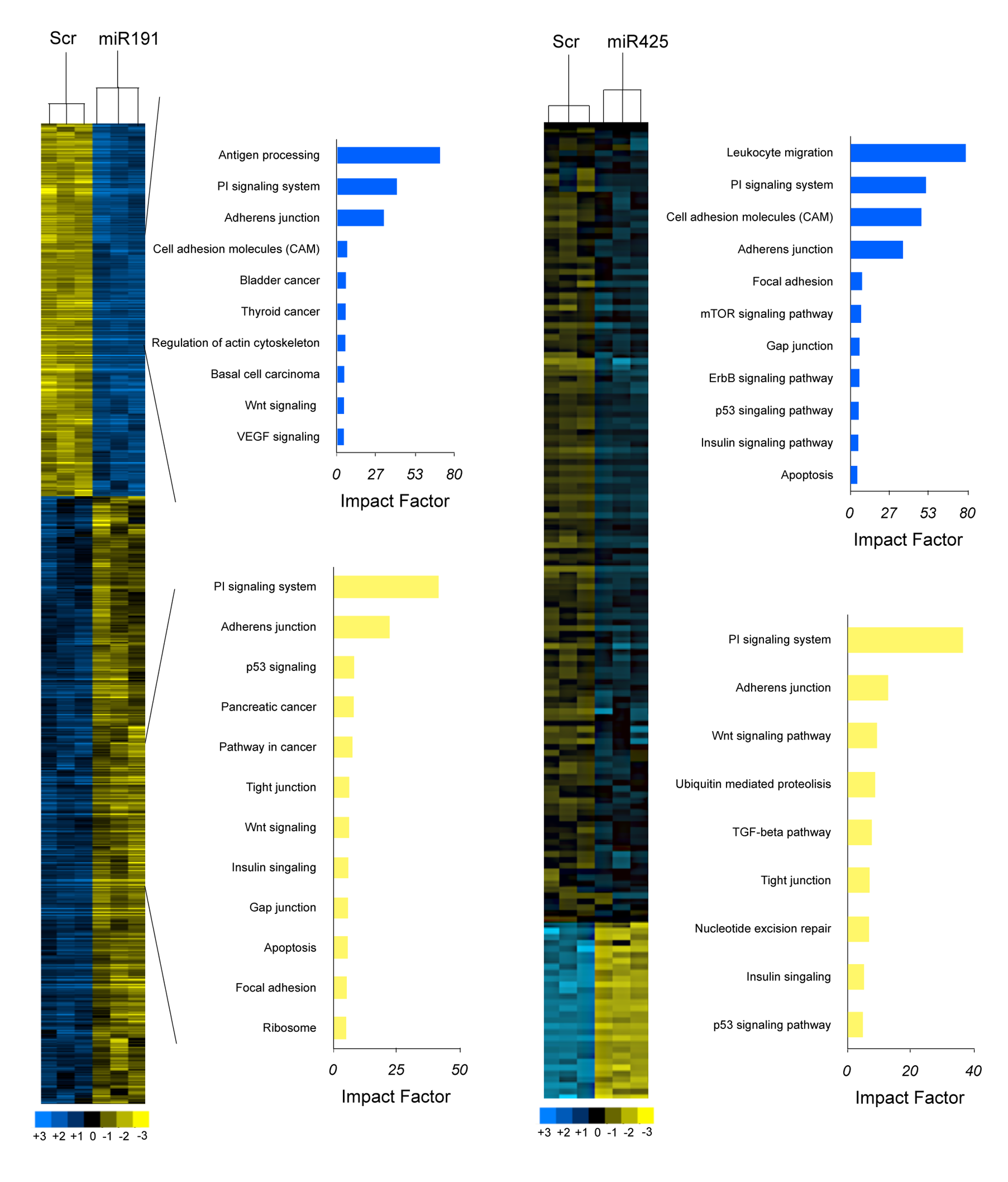

Supplement: Figure S10 — miR-191 and miR-425 signature in aggressive breast cancer cells. miR-191, miR-425 and scrambled control were transfected in MDA-MB-231 and cells were collected 72 h after transfection for genome-wide expression analyses. Differentially expressed genes (fold change >1.2 and p-value<0.001) are represented in the hierarchical tree and the modulated biological pathways are enlisted based on the Impact factor strength of miR-activated (blue) and repressed (yellow) genes. (TIF) [file pgen.1003311.s010.tif]

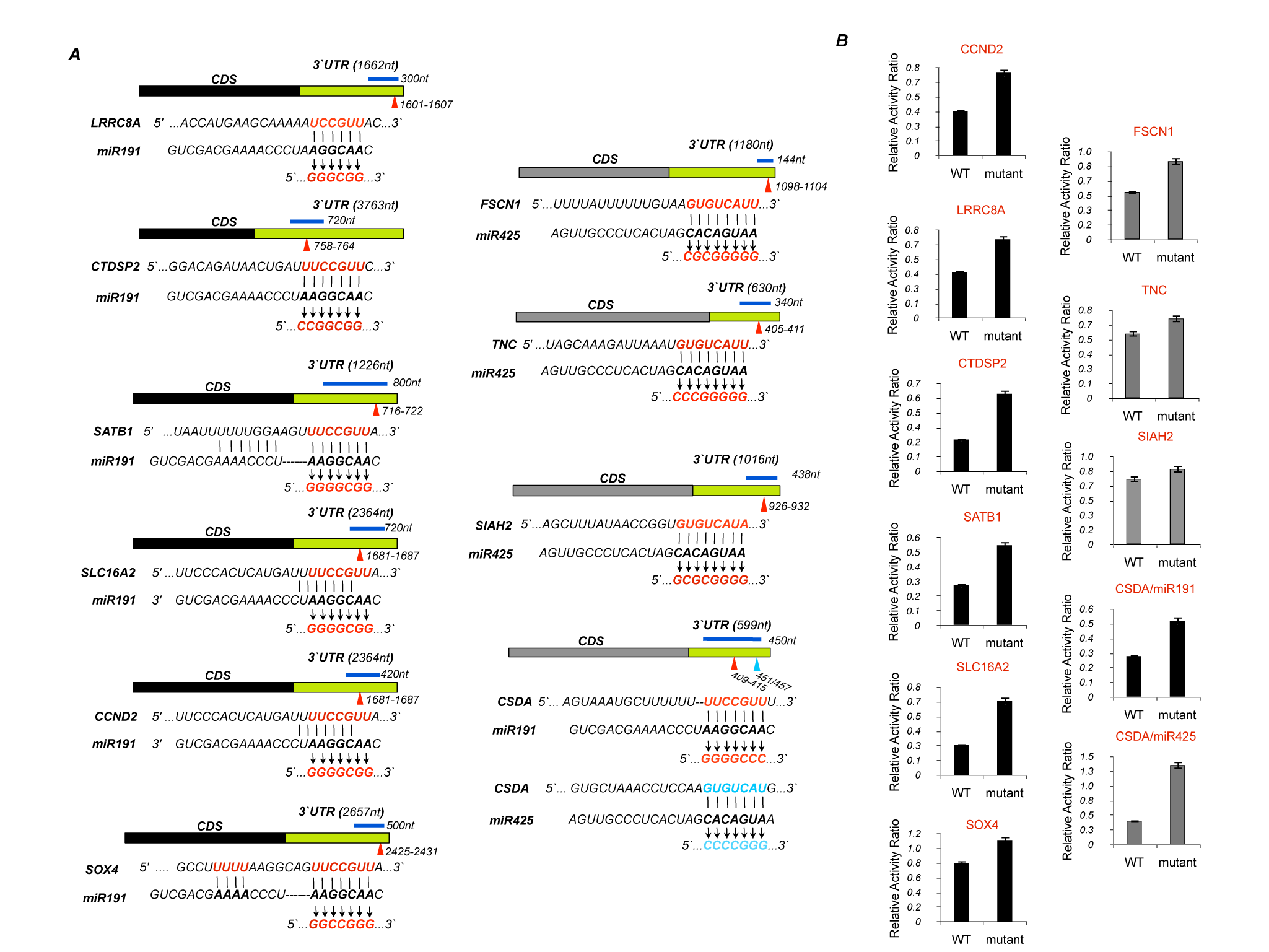

Supplement: Figure S11 — Representation of miR191/425 binding sites of the target genes. (A) Schematic representation of miR191 and miR425 binding sites located in the 3′UTR (in green) of the target genes. The blue line defines the 3′UTR fragment cloned into pGL3 control plasmid and the arrowhead indicates the position of the miRNA binding site, whose sequence is reported below with the mutagenesis strategy used to generate the disruption between miRNA and mRNA of the target gene. (B) Luciferase assays for wildtype and disrupted miRNA binding sites of all target genes with increased luciferase activity after miR191 or miR425 enforced expression on mutated plasmid. Luciferase activity values for wildtype and mutated plasmids are represented as a ratio between the relative luciferase activity of miR-transfected cells with the relative luciferase activity of control-transfected cells. (TIF) [file pgen.1003311.s011.tif]

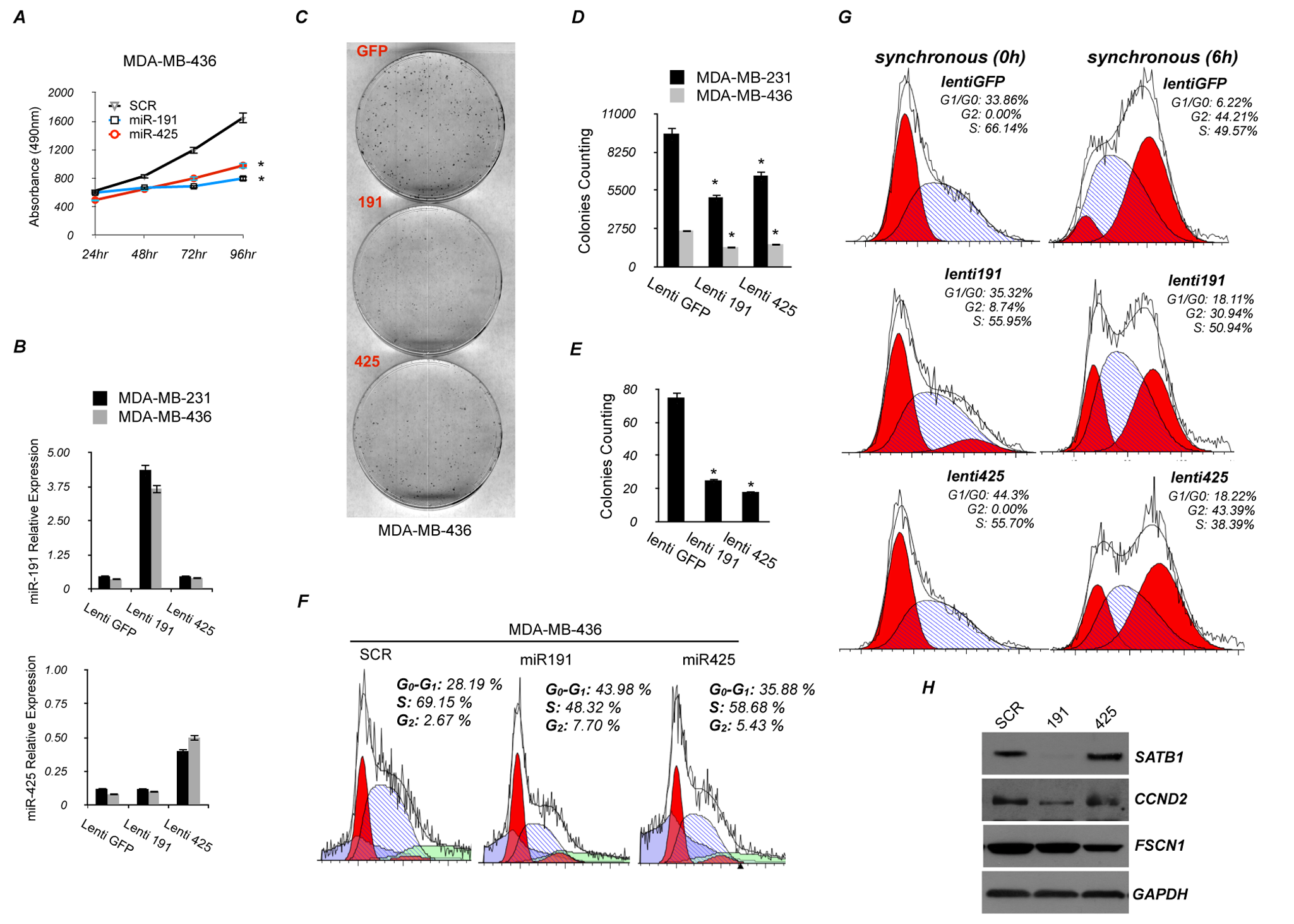

Supplement: Figure S12 — miR-191/425 impair tumorigenicity of aggressive breast cancer cells. (A) MTT assay revealed a reduced growth rate in miR-191 and miR-425 overexpressing MDA-MB-436 cells compared to scrambled control cells. Error bars indicate s.d. and asterisks indicate p-value<0.05. (B) qRT-PCR to verify miR-191 and miR-425 overexpression in lenti-infected MDA-MB-231 or MDA-MB-436. Error bars indicate s.d. (C,D) 2D colonies formation assay in MDA-MB-436 stable cell line expressing miR-191 or miR-425 from lentiviral expression vectors, compared to the corresponding GFP control cells. Colony counting was performed by using the GS-800™ calibrated densitometer. Error bars indicate s.d. and asterisks indicate p-value<0.05. (E) Soft agar assay in which cells were seeded at a density of 4×10^3^ cells per 35-mm dish and cultured in 0.35% soft agar in RPMI 10% FBS at 37°C for 21 days. Colonies were stained with 0.05% crystal violet. Colony numbers in the entire dish were counted by using the GS-800 calibrated densitometer. Error bars indicate s.d. and asterisks indicate p-value<0.05. (F) Cell cycle analyses of MDA-MB-436 transiently-transfected cells. Cells were harvested 72 h following transfection, fixed, stained with propidium iodide and analyzed by flow cytometry. The data obtained were analyzed using ModFit software. Cells in G1 and in G2 phase of cell cycle are reported in red, cells in S phase are indicated with white and blue bars. Flow cytometry plots are representative of three independent experiments. (G) Lenti-viral infected MDA-MB-231 were analyzed by propidium iodide staining after 100 ng/mL nocodazole treatments for 16 h, before cells were released and harvested for FACS analysis. The data obtained were analyzed using ModFit software. Cells in G1 and in G2 phase of cell cycle are reported in red, cells in S phase are indicated with white and blue bars. Flow cytometry plots are representative of three independent experiments. (H) miR-191/425 modulated targets were analyzed by We [file pgen.1003311.s012.tif]

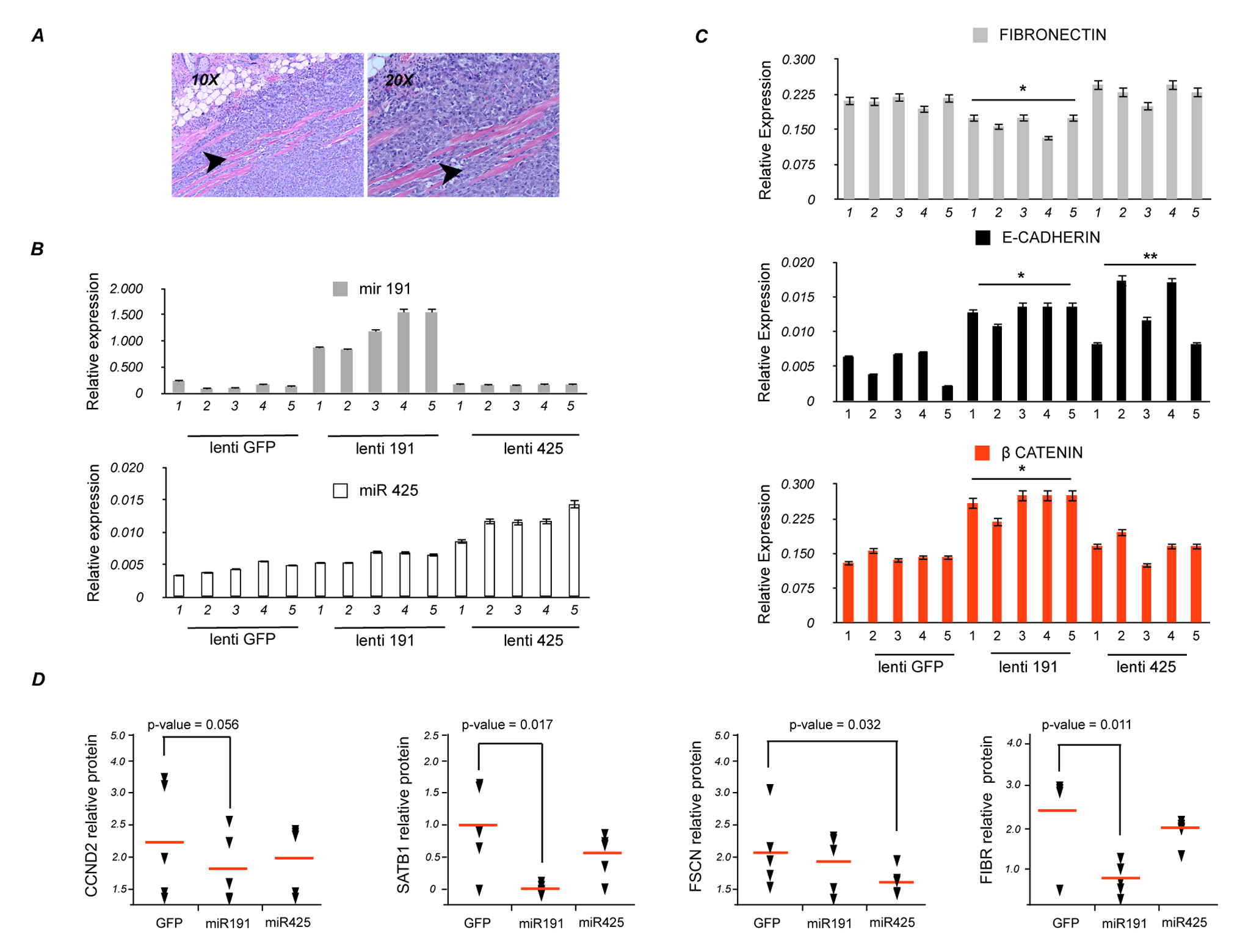

Supplement: Figure S13 — miR-191/425 in vivo effects. (A) Hematoxylin-eosin of subcutaneous MDA-MB-231 lentiGFP-infected tumors. Arrows in panels indicate areas of tumor invasion in the muscle cells of the fibrotic capsule. (B) qRT-PCR to verify the expression of miR-191 and miR-425 in the resected xenografted tumors. (C) Expression levels of fibronectin, e-cadherin and beta-catenin were determined by taqman qRT-PCR in xenografted tumors. Error bars indicate s.d. (* indicates p-value<0.01; ** indicates p-value = 0.011). (D) Densitometric analyses of the Western blots presented in Figure 5H. (TIF) [file pgen.1003311.s013.tif]

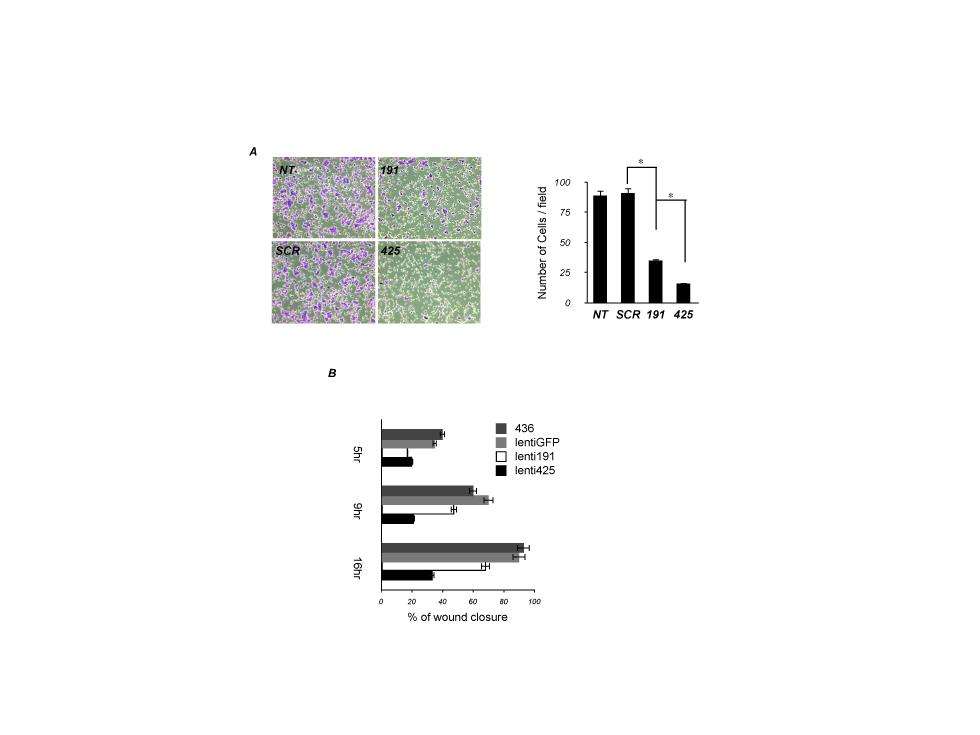

Supplement: Figure S14 — miR-191/425 impair motility of MDA-MB-436 breast cancer cells. (A) Transwell motility assay was performed by plating miR191,-425 and scrambled control transfected MDA-MB-436 cells on inserts. Collected data from three separate experiments performed in triplicate are represented as number of cells per field. (B) Wound healing assay done on the miR-191,-425 stable clones and GPF control cells MDA-MB-436. The diameters of wounds were measured on the microscopic photos at 0, 5 h, 9 h and 16 h after wounding. Changes in wound diameter were computed into percentage to represent wound closure. Error bars indicate s.d. (TIF) [file pgen.1003311.s014.tif]
